# Studies on biotransformation mechanism of *Fusarium* sp. C39 to enhance saponin content of Paridis Rhizoma

**DOI:** 10.3389/fmicb.2022.992318

**Published:** 2022-12-16

**Authors:** Yiyang Chen, Dan Yu, Jinhai Huo, Nannan Huang, Meng Zhang, Xiaowei Du

**Affiliations:** ^1^Key Laboratory of Chinese Materia Medica, Ministry of Education, Pharmaceutical College, Heilongjiang University of Chinese Medicine, Harbin, China; ^2^Institute of Chinese Materia Medica, Heilongjiang Academy of Chinese Medicine Sciences, Harbin, China

**Keywords:** Paridis Rhizoma, strain C39, steroidal saponins, cytotoxicity, UPLC-Q/TOF-MS, transcriptome

## Abstract

Paridis Rhizoma is a natural medicine with strong anti-tumor and anti-inflammatory activities. Our previous research have found that *Fusarium* sp. C39, an endophytic fungus isolated from Dioscorea nipponica which contains the similar chemical components, significantly increased the steroidal saponins content of Paridis Rhizoma by fermentation. In this study, the inhibitory effects of fermentated Paridis Rhizoma extract (PRE) on liver cancer cells (Hepal-6), cervical cancer cells (Hela), and lung cancer cells (A549) were determined to be stronger than that of the unfermented extract. For discovering the fermentation mechanism of PRE with *Fusarium* sp. C39, 36 components with obviously quantitative variations were screened out by UPLC-Q/TOF-MS and 53 key genes involved in the metabolic pathways of steroidal saponins were identified by transcriptome. On the basis of comprehensively analyzing information from the metabonomics and transcriptome, it can be speculated that the increase of spirostanol saponins and nuatigenin-type saponins enhanced the inhibitory effect of fermented PRE on cancer cell proliferation. Under the action of glycosidase, glycosyltransferase, oxidoreductases, and genes involved in sterol synthesis, strain C39 achieved the synthesis of diosgenin and the alteration of configurations, sugar chain and substituent of steroidal saponins. The research suggested a microbial transformation approach to increase the resource utilization and activity of *Paris polyphylla*.

## Introduction

Paridis Rhizoma, the rhizome of the perennial herb *Paris polyphylla* Smith var. *yunnanensis* (Franch.) Hand. Mazz. (PPY) or *Paris polyphylla* Smith var. *chinensis* (Franch.) Hara (PPC), is an indispensable traditional Chinese medicine with the functions of clearing away heat, detoxifying, reducing swelling, and relieving pain. The earliest records of Paridis Rhizoma being applied traced back 2000 years ago to “Shen Nong’s Materia Medica” (China, 2020). At present, Chinese patent medicines that have been widely used clinically with Paridis Rhizoma as the main raw material include “Yunnan Baiyao,” “Chonglou Jiedu Tincture,” “Gongxuening Capsules,” and “Ji Desheng Snake Pills.” Phytochemical and pharmacological studies have shown that PPY and PPC are rich sources of steroidal saponins with significant anti-cancer, anti-inflammatory, hemostasis, immune regulation, and antioxidant effects (Tian et al., 2020; Thapa et al., 2022).

The main distribution areas of *Paris polyphylla* include China, India, Nepal and other countries bordering the Himalayas (Shah et al., 2012). Due to excessive development, illegal collection, open-air grazing, and other human activities, the wild resources of *P. polyphylla* have been severely damaged, and *P. polyphylla* is listed as endangered species, which seriously hinders sustainable resource acquisition (Cunningham et al., 2018; Pokhrel et al., 2019; Chauhan, 2020). At present, the expansion of artificial planting is the main measure to cope with the shortage of resources. But due to the disadvantages of the long growth cycle and low reproduction rate, it is difficult to meet the growing market demand (Yang et al., 2012). Microbial transformation is one of the promising routes to for expanding natural medicine resources. Compared with chemical synthesis, it has higher regioselectivity and stereoselectivity, mild action, high conversion rate, simple operation and low cost (Li et al., 2020). Endophytes are a special class of microorganisms, some of which are capable of producing the same biologically active compounds as the host plants or promoting the accumulation of secondary metabolites in synergy with the host plants. They are of interest as potential resources for producing and transforming natural medicines (Hardoim et al., 2015; Venieraki et al., 2017). However, these microorganisms usually do not have stable production and transformation capabilities when cultured *in vitro*, which limits their application (Venugopalan and Srivastava, 2015). Exploiting the function of valuable endophytes through biotechnology is currently a more useful solution (Kusari et al., 2014a,b; Shankar Naik, 2018). Therefore, exploring the regulatory mechanism of microbial production and the transformation of natural products at the molecular level is of great significance for expanding medical resources.

*Fusarium* *sp*. C39, a filamentous endophytic fungus isolated from *Dioscorea nipponica* that could significantly increase the total saponins content of *D.nipponicae* Rhizoma through fermentation (Du et al., 2013). Further research found that this strain also had similar effects on the saponin content of Paridis Rhizoma. In the liquid fermentation broth after acid hydrolysis treatment, the content of diosgenin increased by 39.00%, while the increase of pennogenin content increased by 288.64%. In this study, we investigated the inhibitory ability of fermented Paridis Rhizoma extract (PRE) on the proliferation of three cancer cell lines, compared the dynamic changes of the saponins content in PRE during fermentation process, and illustrated the differentially expressed genes (DEGs) in strain C39 that regulated the synthesis and transformation of steroidal saponins at the molecular level. The toxic activity of strain C39 on cancer cells and how strain C39 enhances the saponin content of Paridis Rhizoma were initially explored. These studies provide a theoretical basis for the further development of the ability of the strain to transform natural products.

## Materials and methods

### Strains and medium

The strain C39 was isolated from *D. nipponica Makino* and preserved in the Pharmaceutical Laboratory of the Heilongjiang University of Chinese Medicine. The preparation method of the culture medium is as follows: add 200 g of chopped peeled potatoes to distilled water and boil for 30 min, filter and make up the liquid to 800 ml. We added 20 g glucose and 20 g agar, which was packaged and sterilized at high pressure at 121°C for 30 min before use to obtain potato dextrose agar (PDA) medium. Then, we poured the hot medium into Petri dishes and added 35–40 ml of each. The potato dextrose broth (PDB) medium is used to prepare 100 ml seed culture and 150 ml of the drug-containing medium, which lacked the addition of agar during the same preparation process. Dispensed in conical flasks as required and sterilized.

### Preparation of Paridis Rhizoma extract

Paridis Rhizomes were ground into powder and then sieved before use. Two grams of Paridis Rhizomes powder was added with 50 ml of 70% ethanol and reflux extract twice at 85°C for 2 h each time, combined with the filtrate and concentrated to dryness on a rotary evaporator to obtain PRE.

### Liquid-state fermentation of PRE

Strain C39 was activated on a PDA medium for 4–5 days, and a punch with a diameter of 8 mm was used to drill holes at the colony’s edge to obtain fungus cakes. Afterward, each cake was inoculated in 100 ml PDB medium for 3 days to prepare seed cultures and control samples were fermented for 0 days. Subsequently, the PRE was dissolved in 150 ml of PDB medium and sterilized. After cooling, 1.2 ml of seed solution was added to the drug-containing medium and fermented for different times of 3, 5, 7, and 9 days as the sample groups. All groups were incubated at 28°C with a rotation speed of 120 r/min in a shaker table.

### Cell culture and viability assay

As specified in Sections Preparation of Paridis Rhizoma extract and Liquid-state fermentation of PRE, 10 g of Paridis Rhizomes powder was subjected to liquid fermentation for 7 days and served as the sample group, while the unfermented raw medicinal material extract served as the control group. After the samples were concentrated to 20 ml, it was extracted three times with an equal volume of water-saturated n-butanol and the n-butanol layer was retained to prepare a lyophilized powder, which was dissolved in Dimethyl sulfoxide (DMSO) to prepare a 15 mg/ml mother solution, and stored at 4°C. The stock solution was diluted to the desired concentration with serum-free medium, followed by filtration and sterilization.

Hepal-6 liver cancer cells, Hela cervical cancer cells, and A549 lung cancer cells (Heilongjiang University of Chinese Medicine, Pharmacognosy Laboratory Storage) were cultured in the medium supplemented with 10% fetal bovine serum (FBS, Gibco, United States), 100 U/ml penicillin, and 100 μg/ml streptomycin. The medium of Hela and A549 cells is RPMI 1640 (Gibco, US), while the medium of Hepa1-6 cells is Dulbecco’s modified Eagle’s medium (DMEM, Gibco, United States), and all cells were incubated at 37°C and 5% CO_2_. Cells in the exponential growth phase were digested by trypsin and seeded in 96-well plates at 1 × 10^5^ cells/mL density. After overnight incubation, cells were treated with different concentrations of the samples and incubated for another 24 h. Subsequently, the supernatant was discarded, 20 μl of 3-(4, 5-dimethylthiazol-2-yl)-2, 5-diphenyltetrazolium bromide (MTT) was added to each well, and the cells were incubated for 4 h. The supernatant was aspirated, supplemented with 100 μl of DMSO per well, and shaken for 15 min. The OD (optical density) value was measured at 490 nm using a Microplate Reader (Thermo, United States). Cell inhibition ratio (%) = [1-A_Sample_/A_Contro_l] × 100%.

### UPLC-Q-TOF-MS analysis of saponins

From each sample, 110 ml of culture medium was separated and concentrated, and ethanol was added to 80% concentration. After 12 h of precipitation, the filtrate was recovered under reduced pressure, and then the volume was fixed with 80% ethanol and filtered through a 0.22-μm membrane to obtain the test solution for the analysis of the changes in saponin components. For the standards, Polyphyllin I, II, VI, VII, pseudoprotodioscin, diosgenin and pennogenin were accurately weighed and prepared into a mixed reference solution with a mass concentration of 0.1 mg/ml. Metabolite profiling was conducted using a UPLC system (ACQUITY UPLC; Waters, Milford, MA, United States) and hybrid Q-TOF tandem mass spectrometry (Triple-TOF-MS; Triple TOF 5600 system; AB SCIEX, Concord, ON, Canada). Chromatographic separation was performed on an ACQUITY UPLC BEH C18 column (2.1 mm × 100 mm × 1.7 μm; Waters) using mobile phase A (0.1% formic acid in deionized water) and mobile phase B (0.1% formic acid in acetonitrile). Mobile phase B was increased linearly from 5% at 0 min to 40% at 2 min to 100% at 22 min and then held at 5% until 22.1 min. Finally, solvent B was held at 5% until 25 min. The flow rate was maintained at 0.3 ml min^−1^. Mass data acquisition was performed in both positive [electrospray ionization-positive (ESI^+^)] and negative (ESI^−^) modes using the following parameters: Ion spray voltage of 5.5 kV in ESI^+^ and −4.5 kV in ESI^−^; Nebulizer gas (gas 1) of 55 psi; Heater gas (gas 2) of 55 psi; Curtain gas of 35 psi; Turbo spray the temperature of 550°C; Declustering potential of 100 V in ESI^+^ and −100 V in ESI^−^. The collision energy of 35 V in ESI^+^ (−35 V in ESI^−^); Collision energy spread of 15 V. The mass spectrum scanning range was 80–1,500 Da.

### Data processing and multivariate analysis

To study the dynamic changes of steroidal saponins in the sample group and control group. The collected data is imported into the MarkerView (AB SCIEX, United States) software, and the error between the charge-to-mass ratio and the retention time is normalized by the Alignment&Filtering function; the processed data use the principal component analysis (PCA) method for pattern recognition and establishes the model, and the score matrix Scores and loadings are generated separately to obtain different compounds. Then, we used the established MS database of more than 200 species of plants of the genus *Paris L.* and the cleavage law of steroidal saponins, and used Peakview 2.0 software to pass targeted screening and non-target screening methods to infer the possible structure of the different components.

### RNA extraction, RNA-Seq library construction, and sequencing

In a sterile environment, the remaining culture medium of each sample was separated and filtered with gauze, then rinsed with sterilized distilled water and removed excess water from the mycelium, immediately frozen in liquid nitrogen and stored at −80°C. The total RNA of strain C39 was extracted using TRIzol reagent (ThermoFisher, China) following the manufacturer’s protocol. The concentration of the extracted RNA samples was determined using a Nanodrop system (NanoDrop, Madison, United States), and the integrity of the RNA was examined by the RNA integrity number (RIN) using an Agilent 2,100 bioanalyzer (Agilent, Santa Clara, CA, United States). To construct the cDNA library, mRNA was purified by using Oligo (dT)-attached magnetic beads and sheared into short fragments, which served as templates for synthesizing the cDNAs using random hexamer primers. Following end-repair and adaptor ligation, the products were amplified by PCR and purified to create a cDNA library. The constructed libraries were then sequenced by using the BGIseq-500. The RNA Extraction, cDNA library construction and sequencing were performed at BGITech (Shenzhen, China).

### Transcript assembly and annotation

The sequencing data of the adaptor, the low-quality reads, and the unknown bases were filtered with SOAPnuke (v1.4.0), and then clean reads were obtained and stored in FASTQ format for downstream analyses. HISAT2 (V2.1.0; Kim et al., 2015) and Bowtie2 (V2.2.5; Langmead and Salzberg, 2012) were used to map clean readings to the reference genome and align them with the reference coding gene set, respectively, and then the expression level of gene was calculated by RSEM (V1.2.8; Li and Dewey, 2011).

### Functional annotation and enrichment analysis of DEGs

Differential expression analysis was performed using the DESeq2 (Love et al., 2014) with a Q value ≤ 0.05. To take an insight into the change of phenotype, GO^1^ and KEGG^2^ enrichment analysis of annotated different expressed genes was performed by Phyper^3^ based on Hypergeometric test. The significant levels of terms and pathways were corrected by Q value with a rigorous threshold (Q value ≤ 0.05) by Bonferroni. To visualize the results of GO enrichment of DEGs, Cytoscape version 3.6.1 with BiNGO plugin version 3.0.3 (Maere et al., 2005) were used to construct significantly enriched biological networks and output the results as graphs. To predict the functional interaction of proteins, DIAMOND (Buchfink et al., 2021) was used to align the genes to the STRING database, and the network relationship with a score value of ≥300 was screened into Cytoscape to visualize the PPI network, and the cytoHubba plugin was used to screen hub genes to construct a sub-network.

### Validation of transcripts by quantitative qRT-PCR

The reliability of RNA-seq data was validated by qRT-PCR using the AriaMx Real-Time PCR System (Agilent, United States). Primer sequences were designed based on mRNA by Sangon Biotech (Sangon, China) and Takara Bio Inc. (Takara, Japan) using Primer 5 in the laboratory and are shown in Supplementary Table S1. First-strand cDNA was synthesized from the total RNA using PrimeScript™ RT Reagent Kit with gDNA Eraser and then TB Green ® Premix Ex Taq™ П (Takara, Japan) was used for qRT-PCR. The total reaction mixture volume for each reaction was 20 μl, and the amplification program was as follows: 95°C for 30 s, followed by 40 cycles of 95°C for 5 s, and 60°C for 30 s. The tubulin beta (TUBB) (GME8911_g) was selected as the housekeeping gene for the normalization, and three technical replicates were performed for each sample. The relative expression levels for each gene were calculated using the 2^−△△Ct^ method.

## Results

### Inhibitory effects of fermentation products on cancer cells

**Figure 1:**
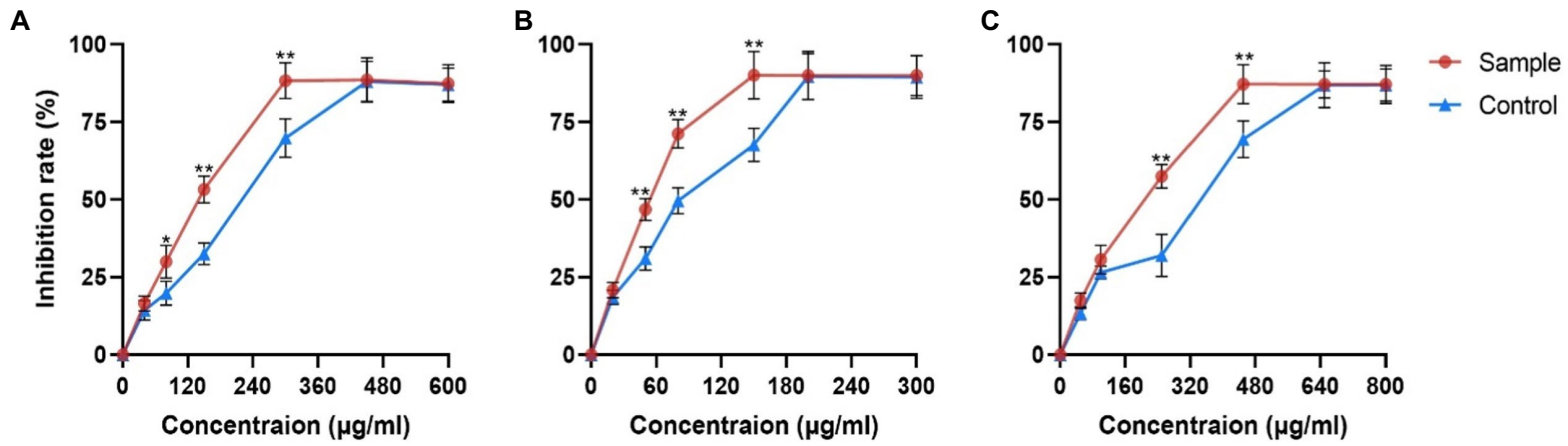
Inhibitory effects of fermentation products on proliferation of different cancer cells. **(A)** Hepa1-6 cells **(B)** Hela cells and **(C)** A549 cells. Values are means ± SD. The asterisk above the line chart denoted statistical significance (*p < 0.05, ** *p* < 0.01, and ns *p* > 0.05).

PRE effectively reduced the viability of three cancer cells (Figure 1). The IC50 values of the control group were 234 μg/ml in Hepa1-6 cells, 97 μg/ml in Hela cells and 315 μg/ml in A549 cells, while the values of the sample group decreased by 29.48, 39.17, and 32.06%, respectively.

### Mass spectrometric fragmentation and chromatographic retention behavior of steroidal saponins in Paridis Rhizoma

**Figure 2:**
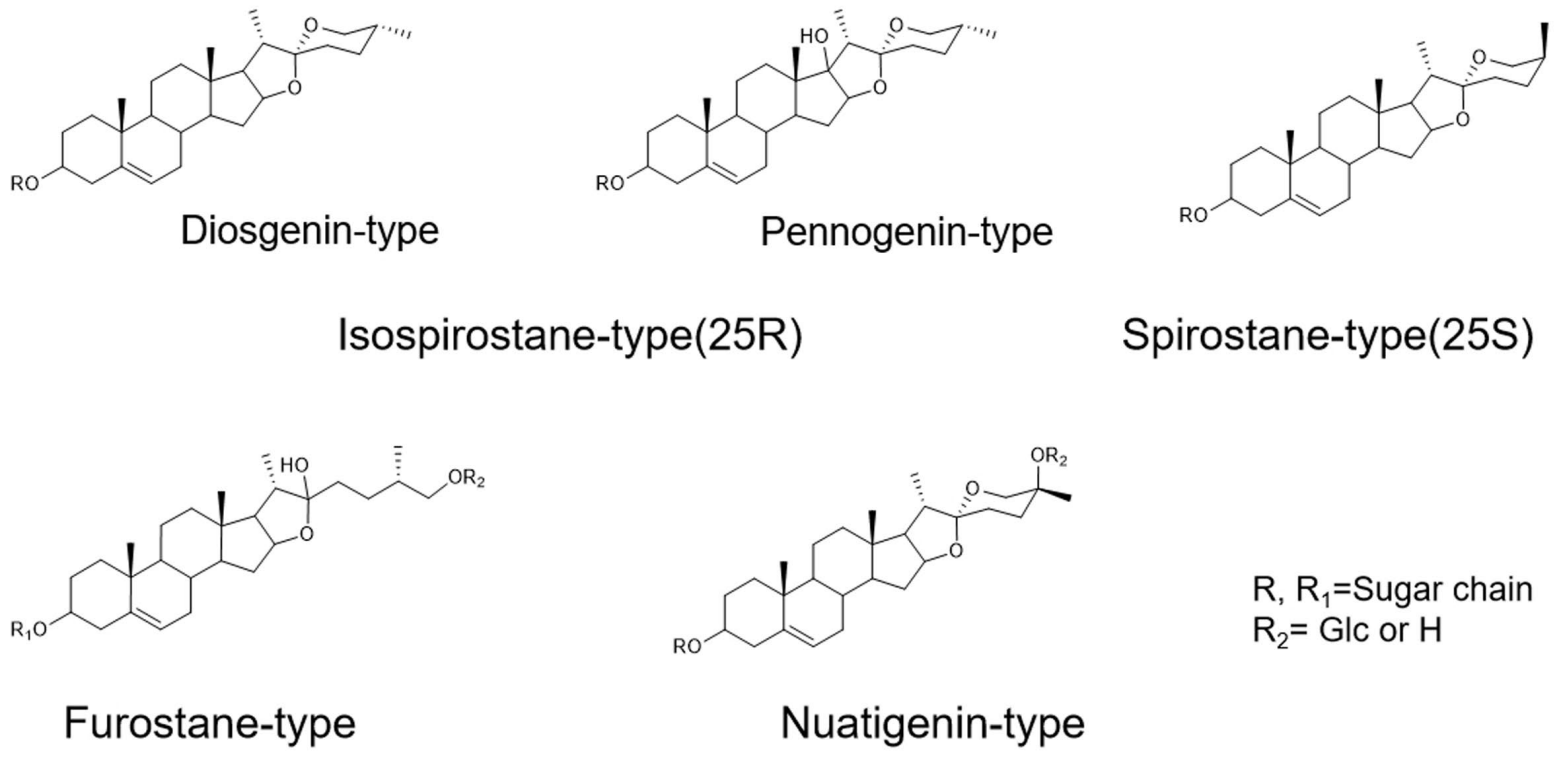
The main configuration of steroidal saponins in *Paris polyphylla*.

**Figure 3:**
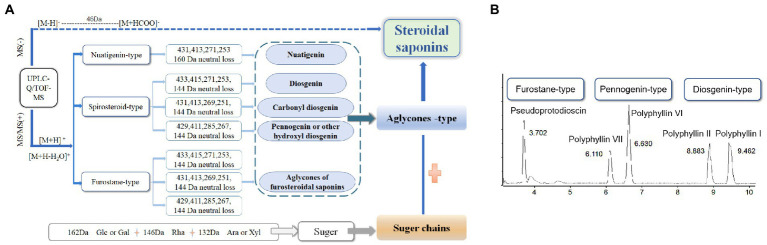
Strategy used for characterizing steroidal saponins in Paridis Rhizoma by HPLC-Q/TOF-MS. **(A)** Chromatographic behavior of steroidal saponins with different configurations. **(B)** BPC chromatograms of UPLC-MS for reference standards at ESI^−^.

To assist in determinating steroidal saponins in PRE, a database containing the reported compound of the plants of *Paris L.* was first constructed (Zhao et al., 2009; Qin et al., 2012, 2016; Wu et al., 2012a,b; Ling et al., 2015), and according to the configuration of C-25 and the state of the F ring, the main steroidal saponins were classified into four types: isospirostane-type (25R), spirostane-type (25S), nuatigenin-type, and furostane-type. Among them, isospirostane-type saponins occupy a major composition, mainly including diosgenin-type and pennogenin-type saponins (Figure 2). Based on the relevant reports on the fragmentation mechanism of steroidal saponins of the *Paris polyphylla*, the characteristic fragmentation patterns were summarized, and a strategy for characterizing the structure of steroidal saponins was proposed and further verified by five reference standards, including two diosgenin-type saponins, two pennogenin-type saponins, and one furostane-type saponins (Figure 3). Generally, the molecular mass and formula could be obtained from the [M + H]^+^, [M + Na]^+^ [M + HCOO]^−^ and [M-H]^−^ ions.

### Qualitative analysis by UPLC-Q/TOF-MS

**Figure 4:**
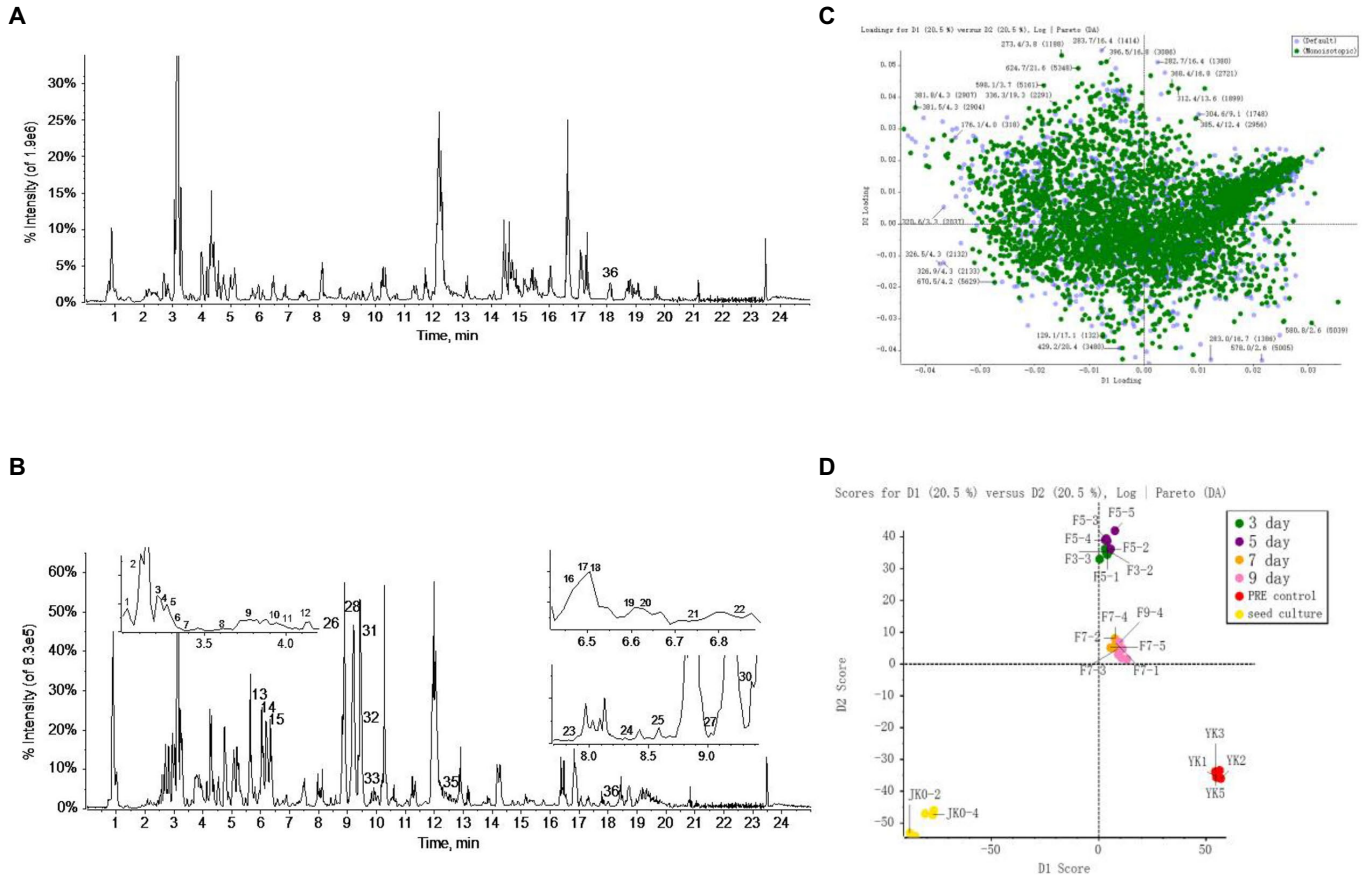
The screening of differential saponins during liquid fermentation of PRE by strain C39 at ESI^+^. **(A)** BPC chromatograms of UPLC-MS for sample group and **(B)** seed culture solution group. **(C)** PCA scores plot and **(D)** loadings plot of all groups.

**Figure 5:**
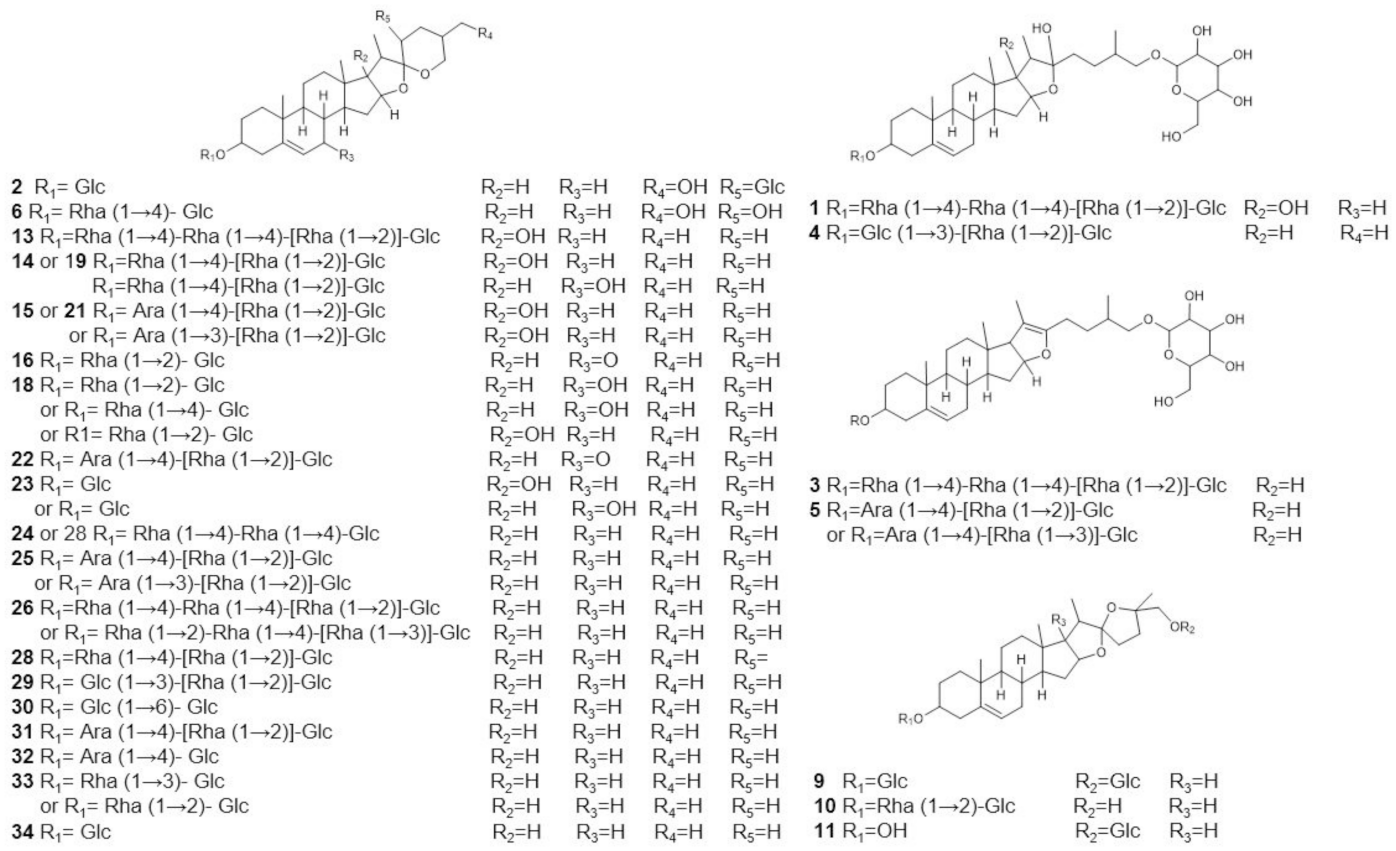
Possible structure of labeled saponins based on UPLC-Q/TOF-MS.

The metabolome data of fermentation samples at days 3, 5, 7, and 9, as well as control samples were collected by UPLC-Q/TOF-MS, and the base peak chromatograms (BPC) in the positive mode were shown in Figures 4A,B. Combined with the method of principal component analysis (PCA), the data were pattern recognized, and a model was established to produce scores plot and loadings plot, respectively (Figures 4C,D). The scores plot showed that the six groups of samples were divided into 4 four types, and the control group differed significantly from the fermentation group. However, no distinction occurred between 3 days and 5 days of fermentation and between 7 days and 9 days of fermentation. In the loadings plot, the distance between each point and the origin represented the contribution to the typing. The ions represented by the farther away points were the focus of the analysis, and thus a total of 36 labeled metabolites including seven furostane-type saponins (peaks 1, 3–8), 23 spirostanol saponins (peaks 2, 13–34), four nuatigenin-type saponins (peaks 9–12) and two glycosides (Peaks 35, 36), were screened out as the identification components (Figure 5; Table 1).

**Table 1:** Data of the identified differential 36 compounds by UPLC/Q-TOF-MS.

Peak NO.	t_R_/min	Selected ion	Measured Mass(m/z)	Error (ppm)	m/z	Main secondary fragment ions (MS/MS) and their sources	Identification
1	2.99	[M + H -H_2_O]^+^	1193.5985	−2.51	C_57_H_94_O_27_	1193.5876,1013.5273,867.4690,721.4110,593.3650,431.3141,,413.3042,251.1800	Th
2	3.1	[M + H]^+^	753.4029	4.25	C_38_H_56_O_15_	753.4029,591.3508,573.3425,447.2375,429.1919,411.2897,267.1746,251.1793	Chonglouoside SL-17
3	3.21	[M + H]^+^	1177.5975	2.63	C_57_H_92_O_25_	1177.5924,1015.5408,869.4841,723.4268,577.3702,415.3189,397.3091,271.2053,253.1225,	Pseudoproto-Pb
4	3.25	[M + H -H_2_O]^+^	1047.5306	6.68	C_51_H_82_O_22_	1047.5362,885.4844,741.3654,577.3734,415.3202,397.3091,271.2058,253.1952	Progracillin
5	3.28	[M + H]^+^	1017.5236	3.44	C_50_H_82_O_22_	1017.5264,855.4728,711.3587,577.3732,415.3205,397.3100,271.2054,253.1946	Parisyunnanoside B or 26-O-β-D-glc-(25R)-5,20 (22)-diene-furost-3β,26-diol-3-O-α-L-ara-(1 → 4)-[α-L-rha-(1 → 2)]-β-D-glc
6	3.31	[M + H -H_2_O]^+^	737.4069	5.97	C_39_H_62_O_14_	737.4106,719.4003,575.3545,539.3386,429.2977,411.2908,271.2043,251.1790	Chonglouoside SL-2
7	3.38	[M + H]^+^	593.3702	−2.19	C_33_H_52_O_9_	593.3658,431.3147,413,3,041,271.2044,253.1946	
8	3.58	[M + H -H_2_O]^+^	721.4154	1.25	C_39_H_62_O_13_	721.4154,575.3592,413.3005,395.2962,269.1909,251.1788	
9	3.77	[M + H]^+^	755.4198	2.65	C_39_H_62_O_14_	755.4207,595.3080,433.2575,413.3043,395.2938,271.2042,253.1944	Chonglouoside SL-11
10	3.94	[M + H]^+^	739.4236	4.46	C_39_H_62_O_13_	739.4249,593.3628,579.3147,431.3166,413.3044,271.2052,253.1944	Nuatigenin 3-O-α-L-rha-(1 → 2)-β-D-glc or Isonuatigenin 3-O-α-L-rha(1 → 2)- β-D-glc
11	3.96	[M + H]^+^	593.3663	4.38	C_33_H_52_O_9_	593.3644,433.2561,431.3159,413.3063,271.2048,253.1947	Chonglouoside SL-9
12	4.08	[M + H]^+^	871.4691	0.00	C_44_H_70_O_17_	871.4655,739.4223,711.3554,593.3661,431.3145,413.3020,271.2053,253.1944	
13	5.97	[M + H -H_2_O]^+^	1013.5296	2.57	C_51_H_82_O_21_	1013.5266,867.4702,721.4135,575.3550,413.3042,395.2944,269.1902,251.1793	Polyphyllin VII
14	6.13	[M + H -H_2_O]^+^	867.4724	2.19	C_45_H_72_O_17_	867.4682,721.4130,575.3552,413.3036,395.2934,269.1892,251.1789	Pennogenin-3-O-α-L-rha(1 → 2)-[α-L-rha(1 → 4)]-β-D-glc or Disoseptemloside E
15	6.27	[M + H -H_2_O]^+^	853.4567	2.23	C_44_H_70_O_17_	853.4548,721.4138,575.3558,413.3030,395.2937,269.1889,251.1790	Paris saponin H
16	6.47	[M + H]^+^	737.4067	6.24	C_39_H_62_O_14_	737.4096,593.2948,447.2368,429.2995,411.2884,285.1842,267.1731,	(3β,25R)-3-hydroxyspirost-5-en-7-one-3-O-α-L-rha-(1 → 2)-β-D-glc
17	6.51	[M + H]^+^	591.3493	6.76	C_33_H_52_O_10_	591.3507,447.2360,429.3005,411.2894,267.1733	
18	6.52	[M + H]^+^	721.4134	4.02	C_39_H_62_O_13_	721.4114,575.3588,413.3030,395.2935,269.1890,251.1787,	Tb or Disoseptemloside D or Sansevierin A
19	6.61	[M + H]^+^	867.4697	5.30	C_45_H_72_O_17_	867.4686,721.4021,575.3516,413.3036,395.2927,269.1903,251.1774,	Pennogenin-3-O-α-L-rha (1 → 2) -[α-L-rha(1 → 4)]-β-D-glc or Disoseptemloside E
20	6.64	[M + H]^+^	883.4669	2.60	C_45_H_72_O_18_	883.4609,737.4087,591.3521,429.2979,411.2873,285.1838,267.1735	
21	6.74	[M + H -H_2_O]^+^	853.4560	3.05	C_44_H_70_O_17_	853.4538,721.4162,575.3519,413.3933,395.2936,269.18861,251.1799	Paris saponin H
22	6.85	[M + H]^+^	869.4537	−0.23	C_44_H_70_O_18_	869.4536,721.3788,591.3515,447.2342,429.2988,411.2880,285.1839,267.1839	(3β,25R)-3-hydroxyspirost-5-en-7-one-3-O-α-L-ara-(1 → 4)-[α-L-rha-(1 → 2)]-β-D-glc
23	7.83	[M + H]^+^	593.3657	5.39	C_33_H_52_O_9_	593.3646,449.2534,413.3054,287.1999,269.1887,251.1785	(3β,17α,25R)-spirost-5-ene-3,17-diol 3-O-β-D-glc or Chonglouoside SL-1
24	8.24	[M + H]^+^	869.4867	3.57	C_45_H_72_O_16_	869.4852,725.3711,577.3716,415.3204,397.3085,271.2053,253.1944	Diosgenin 3-O-α-L-rha-(1 → 4)-α-L-rha-(1 → 4)-β-D-glc
25	8.57	[M + H]^+^	855.4729	1.52	C_44_H_70_O_16_	855.4703,723.4268,711.3572,577.3726,415.3189,397.3081,271.2053,253.1946	Pa or Polyphyllin D
26	8.82	[M + H]^+^	1015.5443	3.35	C_51_H_82_O_20_	1015.5431,869.4884,723.4300,577.3729,415.3202,397.3097,271.2058,253.1951	Polyphyllin II
27	9.05	[M + H]^+^	869.4894	0.46	C_45_H_72_O_16_	869.4830,725.3695,577.3696,415.3188,397.3084,271.2050,253.1943	Diosgenin 3-O-α-L-rha-(1 → 4)-α-L-rha-(1 → 4)-β-D-glc
28	9.16	[M + H]^+^	869.4860	4.37	C_45_H_72_O_16_	869.4860,725.3714,577.3717,433.2574,415.3191,387.3085,271.2046,253.1940	Dioscin
29	9.2	[M + H]^+^	885.4836	1.36	C_45_H_72_O_17_	885.4777,723.4262,577.3685,431.3143,415.3179,397.3083,271.2045,253.1936	Gracillin or (3β,25R)-spirost-5-en-3-ol 3-O-β-D-glc-(1 → 6)-[α-L-rha-(1 → 2)]-β-D-glc
30	9.25	[M + H]^+^	739.4211	7.84	C_39_H_62_O_13_	739,4,232,595.3058,577.3770,415.3173,397.3084,271.2046,253.1934	(3β,25R)-spirost-5-en-3-ol 3-O-β-D-glc-(1 → 6)-glc
31	9.4	[M + H]^+^	855.4722	2.34	C_44_H_70_O_16_	855.4665,723.4272,711.3534,577.3695,415.3185,397.3080,271.2046,253.1940	Polyphyllin I
32	9.47	[M + H]^+^	709.4130	4.65	C_38_H_60_O_12_	709.4141,565.2999,433.2596,415.3199,397.3092,271.2053,253.1950	Diosgenin-3-O-α-L-ara-(1 → 4)-β-D-glc
33	9.93	[M + H]^+^	723.4300	2.63	C_39_H_62_O_12_	723.4287,579.3138,415.3192,397.3086,271.2042,253.1943	Progenin II or Disoseptemloside or Eprosapogenin A of dioscin or polyphyllin V
34	10.26	[M + H]^+^	577.3700	6.93	C_33_H_52_O_8_	577.3721,433.2577,415.2438,397.3085271.2055,253.1948	Polyphyllin A
35	12.78	[M + H -H_2_O]^+^	413.3035	5.08	C_27_H_42_O_4_	413.3044,395.2929,269.1895,251.1801,213.1625	Pennogenin
36	18.11	[M + H -H_2_O]^+^	415.3204	1.93	C_27_H_42_O_3_	415.3189,379.3026,283.2408,271.2044,253.1942,213.1651	Diosgenin

**Figure 6:**
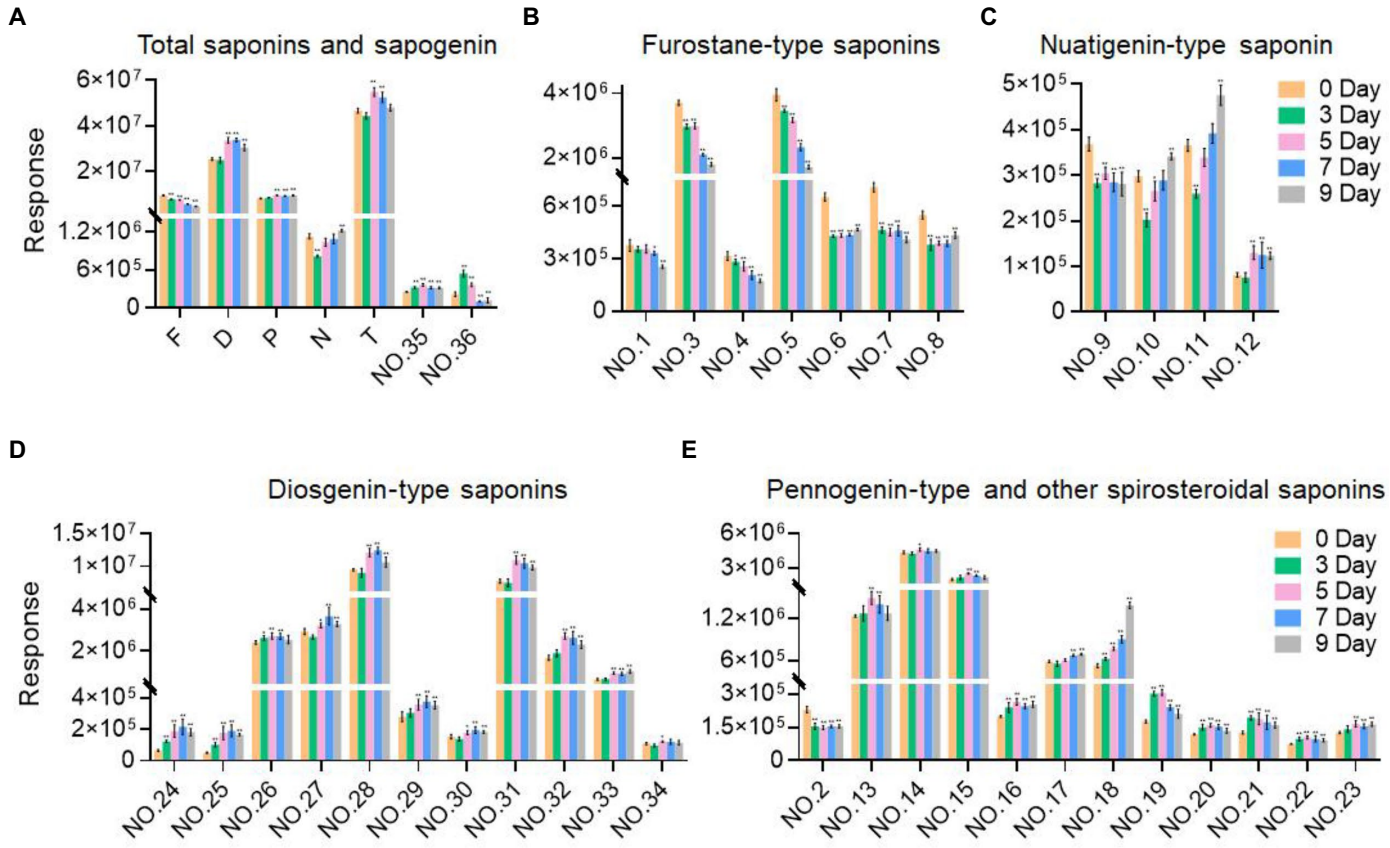
Variation trend of the response values of the selected components with the fermentation time. **(A)** Total saponins and sapogenin. **(B)** Furostane-type saponins. **(C)** Nuatigenin-type saponins. **(D)** Diosgenin-type saponins. **(E)** Pennogenin-type and other spiosteroidal saponins. Values are means ± SD. The asterisk above the bar chart denoted statistical significance (*p < 0.05, **p < 0.01, and ns *p* > 0.05).

As the fermentation time increased, the content of these labeled steroidal saponins showed different changing trends. As a whole, the content of total saponins was first decreasing, then increasing, and then decreasing, and reached the maximum in 5 days of fermentation (Figure 6A). For furostane-type saponins, the content showed an obvious continuous decreasing trend (Figure 6B), while the content of spirostanol saponins generally showed the tendency of first increasing and then decreasing. The content of nuatigenin-type saponins displayed a trend of first decreasing and then increasing (Figure 6C). For diosgenin-type saponins, the content of peaks 26 and 31–34 reached the maximum at 5 days of fermentation, while the peaks 24, 25, 27, 28, 29, and 30 reached the maximum content in fermentation to 7 days (Figure 6D). Among them, the content of peaks 27, 28, 30, 31, and 34 decreased significantly after 3 days of fermentation. According to Figure 6E, pennogenin-type saponins and some saponins with hydroxyl substituents on diosgenin have similar cleavage rules, including peaks 13–15 18, 19, 21, and 23. The content of peaks 19 and 21 reached the maximum at 3 days of fermentation, while the peaks 13, 14, 15, and 23 reached the maximum content at 5 days, and peak 18 showed a continuous upward trend. Peaks 16, 17, 20, and 22 are saponins substituted with carbonyl groups on diosgenin. Peaks 16, 20, and 22 reached the maximum at 3 days of fermentation and peak 17 showed a continuous upward trend. Peak 2 was diosgenin with a hydroxyl group attached to the C23 position, and its content decreased significantly after 3 days of fermentation. In addition, three glycosides were screened. By comparing with the standards, it was determined that peak 35 was pennogenin and the peak 36 was diosgenin, both of which increased first and then decreased (Figure 6A). In the seed culture solution, diosgenin was determined. However, the content of these components varied greatly among parallel samples.

### Transcriptome sequencing, assembly, and annotation

**Figure 7:**
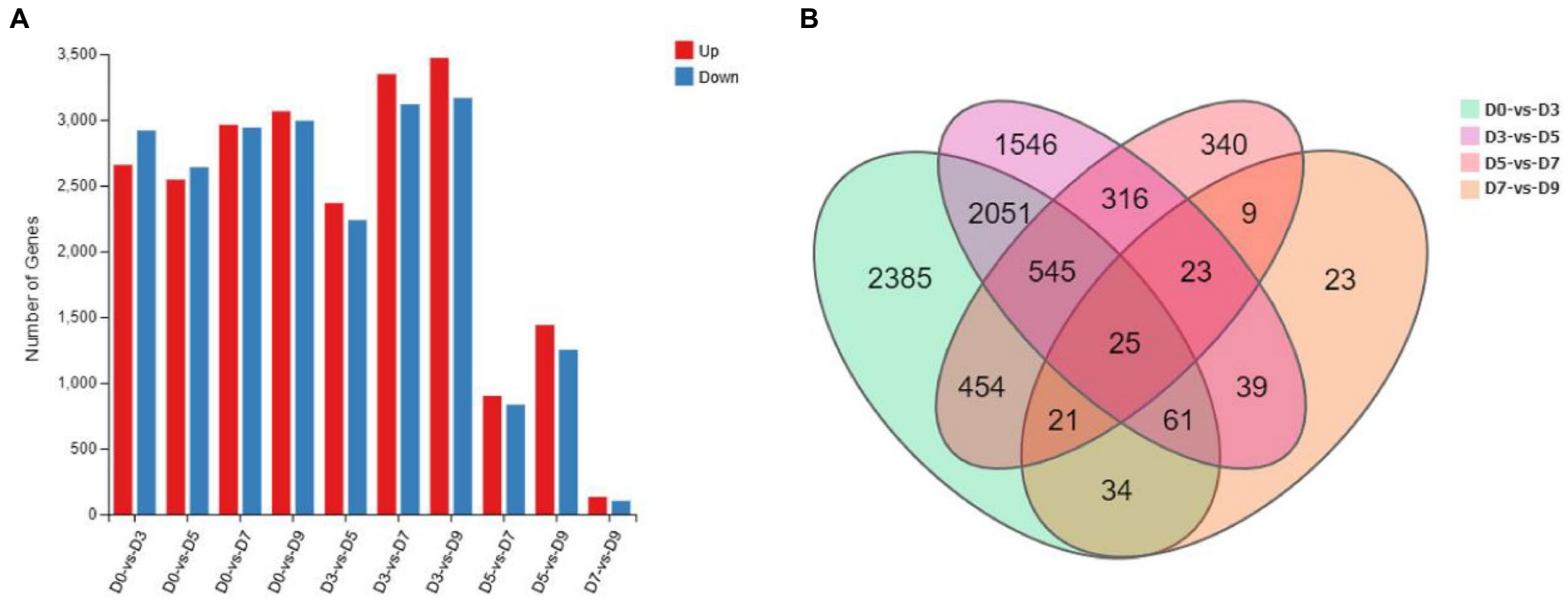
Statistics of the number of DEGs. **(A)** The number of up-down regulated DEGs. **(B)** Venn diagram of DEGs of D0-*vs*-D3, D3-*vs*-D5, D5-*vs*-D7, and D7-*vs*-D9.

A total of 25 mycelial samples at all fermentation time points (0, 3, 5, 7, and 9 days) were tested using the DNBSEQ platform, and each sample produced 6.42Gb data on average. The average comparison rate of the sample comparison genome was 93.78%; the total number of genes detected was 13,481, of which 13,407 were known genes and 74 new genes were predicted; a total of 5,271 new transcripts were detected, of which 3,161 belonged to new alternative splicing subtypes of known protein-coding genes, 74 belong to new protein-coding gene transcripts, and the remaining 2036 belong to long-chain non-coding RNAs. The number of DEGs is shown in Figure 7.

### GO and KEGG analysis

In the early stage, our research group has completed the genome sequencing of the C39 strain; all raw sequencing data were deposited in the NCBI database with the accession numbers JAGFMC000000000, and used to annotate the functional information of the transcripts (Ding et al., 2022).

**Figure 8:**
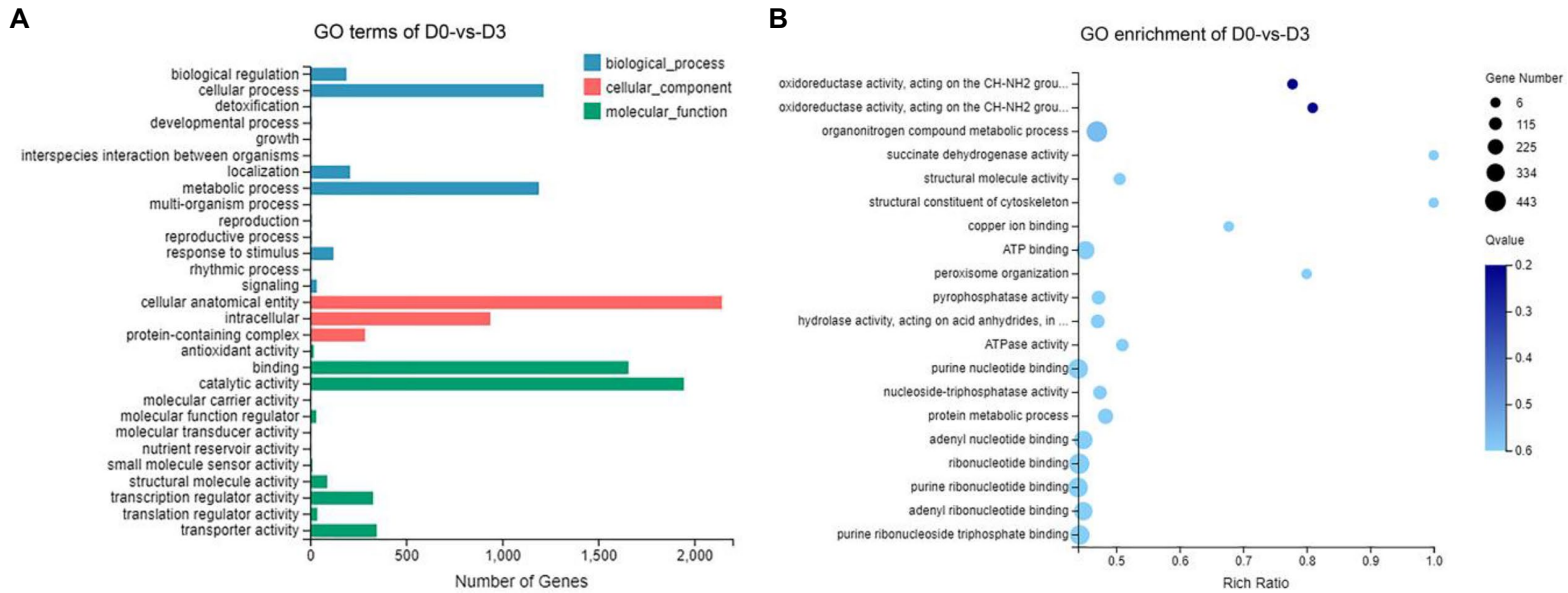
GO analysis of DEGs during the fermentation of D0-*vs*-D3. **(A)** The GO terms analysis and **(B)** GO enrichment analysis of top 20 with the smallest Q value.

Gene Ontology (GO) assignments were used to classify the functions of the DEGs, and the assigned DEGs were divided into three functional categories: molecular functions (MF), cellular components (CC), and biological processes (BP), which were further divided into more detailed subcategories (Figure 8A; Supplementary Figure S1). The number of DEGs of D0-*vs*-D3, D3-*vs*-D5, D5-*vs*-D7 and D7-*vs*-D9 involved in molecular function (MF) was the largest, followed by cellular_component (CC) and biological_process (BP). In the MF category, “catalytic activity” was the most abundant category, followed by “binding.” “transporter activity” and “transcription regulator activity” were similar. In the CC category, “cellular anatomical entities” occupied the largest proportion, and followed by “intracellular” and “protein-containing complexes.” In the BP category, the most abundant GO terms were “metabolic process” and “cellular process,” followed by “biological regulation” and “localization.” The GO enrichment analysis classified the DEGs according to the GO annotation results. D0-*vs*-D3, D3-*vs*-D5, D5-*vs*-D7, and D7-*vs*-D9 were, respectively, assigned 3,033, 2,918, 1888, and 569 GO terms, which contained 3,890, 3,297, 1,244, and 146 DEGs. The GO enrichment of DEGs is shown in Figure 8B; Supplementary Figure S2.

**Figure 9:**
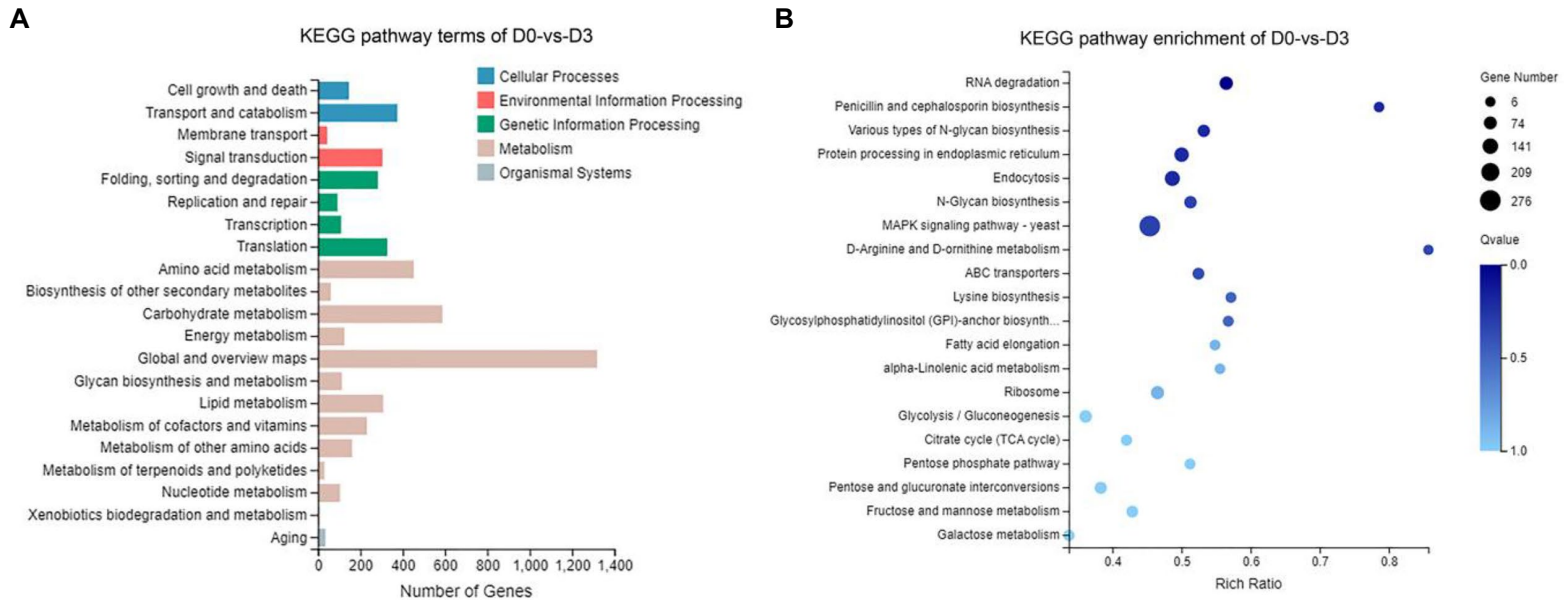
KEGG analysis of DEGs during the fermentation of D0-*vs*-D3. **(A)** The KEGG pathway terms analysis and **(B)** KEGG pathway enrichment analysis of top 20 with the smallest Q value.

**Figure 10:**
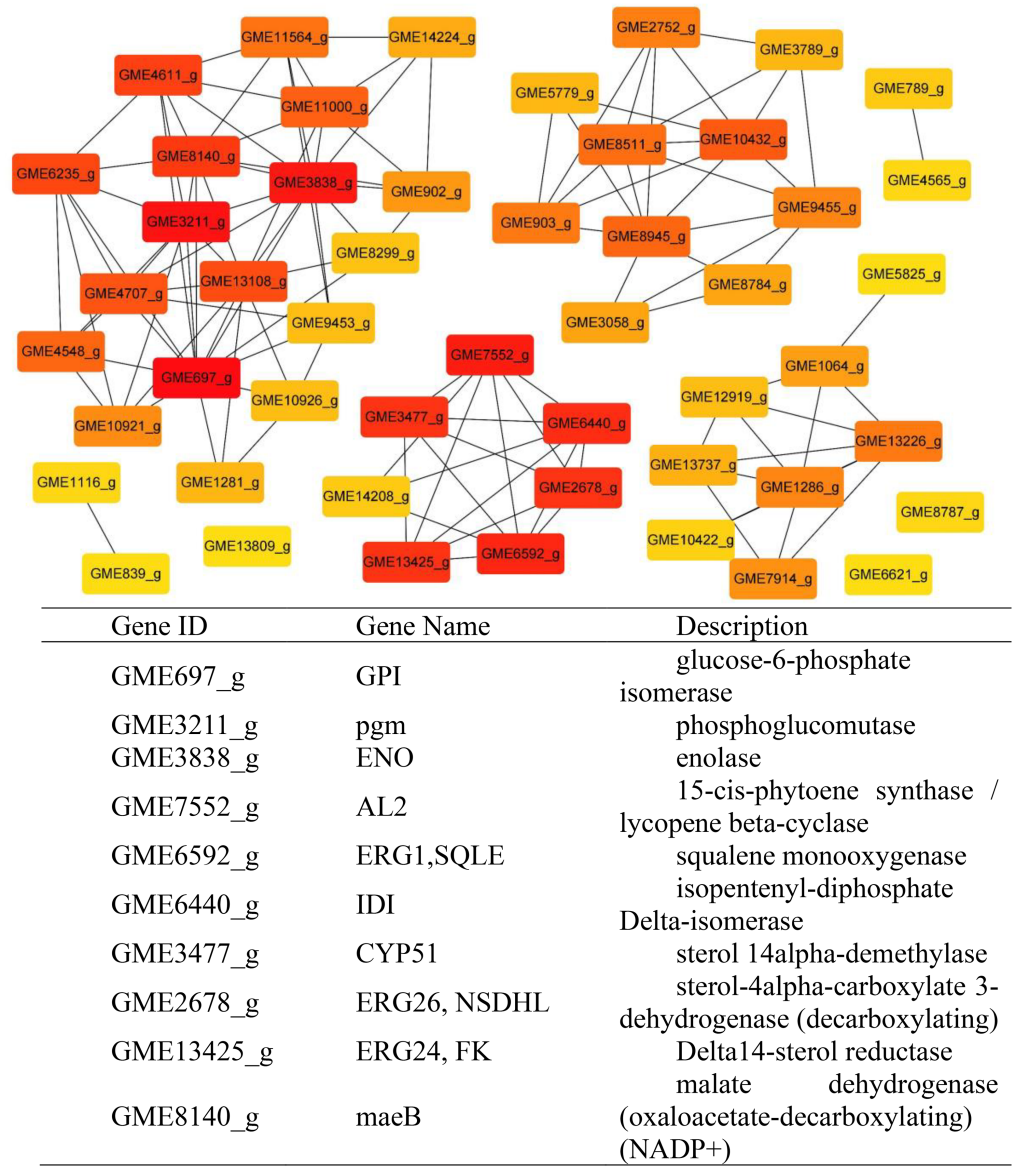
Protein–Protein Interaction network. Dark notes represent hub genes.

According to the results of KEGG, a total of 2,594, 2,162, 809, and 116 DEGs could be aligned to the KEGG pathways teams in D0-*vs*-D3, D3-*vs*-D5, D5-*vs*-D7, and D7-*vs*-D9, respectively. The DEGs involved in KEGG pathway teams were divided into five categories: “Cellular processes,” “Environmental information processing,” “Genetic information processing,” “Metabolism,” and “Organism systems” (Figure 9A; Supplementary Figure S3). The KEGG pathway enrichment of DEGs is shown in Figure 9B; Supplementary Figure S4. Among them, DEGs were significantly concentrated in “Metabolism.” Under this group, the most relevant pathways to the synthesis of steroidal saponins were “Steroid biosynthesis” and “Terpenoid backbone biosynthesis,” which belong to the teams “Lipid metabolism” and “Metabolism of terpenoids and polyketides.” In addition, “Glycan biosynthesis and metabolism” and “Carbohydrate metabolism” were also related to this process. A protein–protein interaction (PPI) network was constructed utilizing DEGs included in these teams, and Cytoscape-cytoHubba was used to screen out the top 50 hub genes and construct a sub-network (Figure 10). The proteins encoding genes glucose-6-phosphate isomerase (GPI), phosphoglucomutase (pgm), enolase (ENO), 15-cisphytoene synthase / lycopene beta-cyclase (AL2), squalene monooxygenase (ERG1; SQLE), isopentenyl-diphosphate Delta-isomerase (IDI), sterol 14alpha-demethylase (CYP51), sterol-4alph-acarboxylate 3-dehydrogenase (decarboxylating) (ERG26; NSDHL), delta14-sterol reductase (ERG24; FK), and malate dehydrogenase (oxaloacetate-decarboxulating) (NADP+) (maeB) may play an important role in increasing saponin content of Paridis Rhizoma by strain C39. Among all KEGG pathway terms, “Pentose phosphate pathway,” “RNA degradation,” “Ribosome,” and “mRNA surveillance pathway” were considered as significantly differential pathways (Q < 0.05).

### Metabolic pathway analysis of candidate genes involved in steroidal saponin synthesis

**Figure 11:**
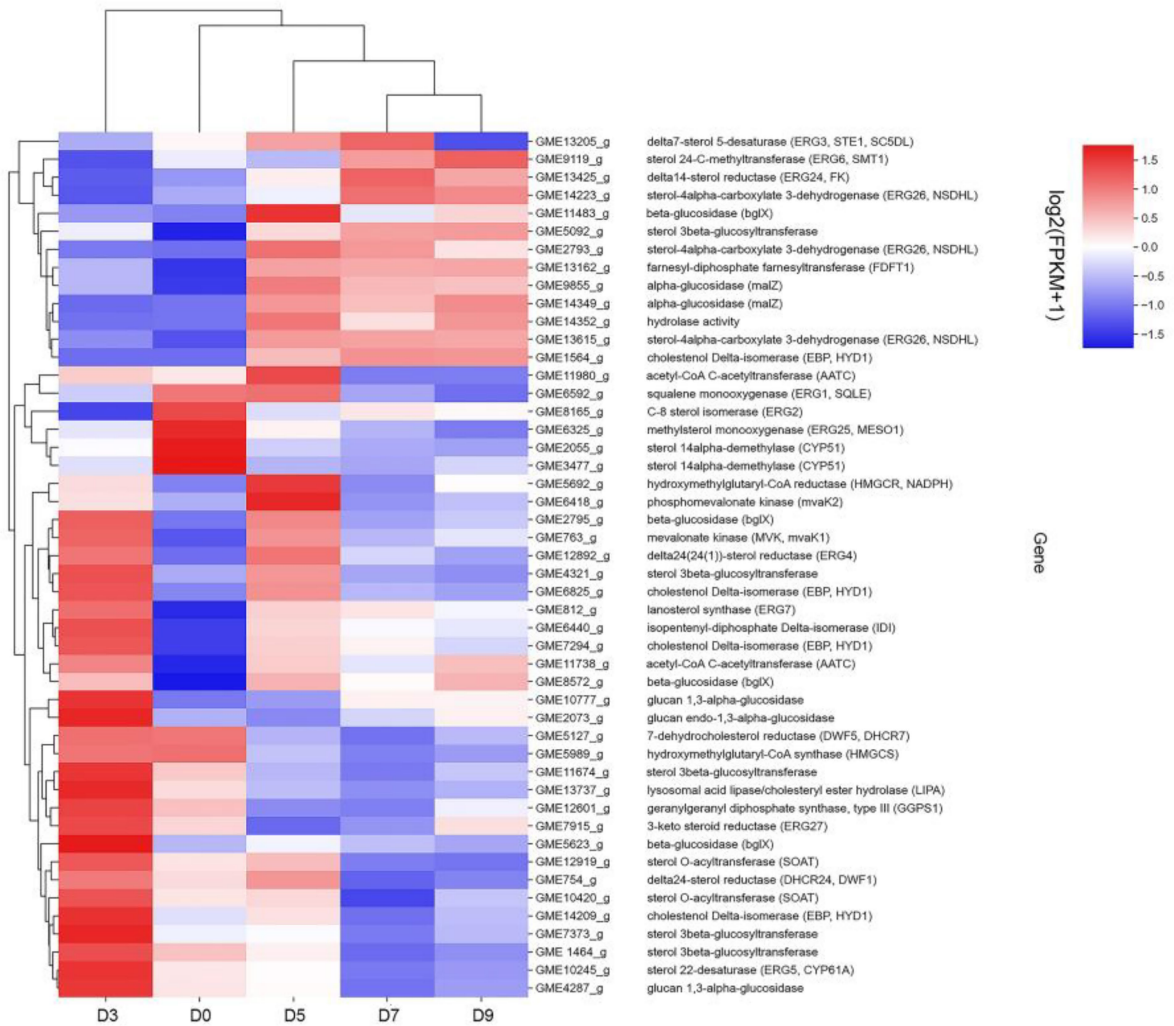
DEGs expression heatmap related to the synthesis and transformation of steroidal saponins by strain C39. The color from blue to red indicated log2 (FPKM +1) from low to high.

**Figure 12:**
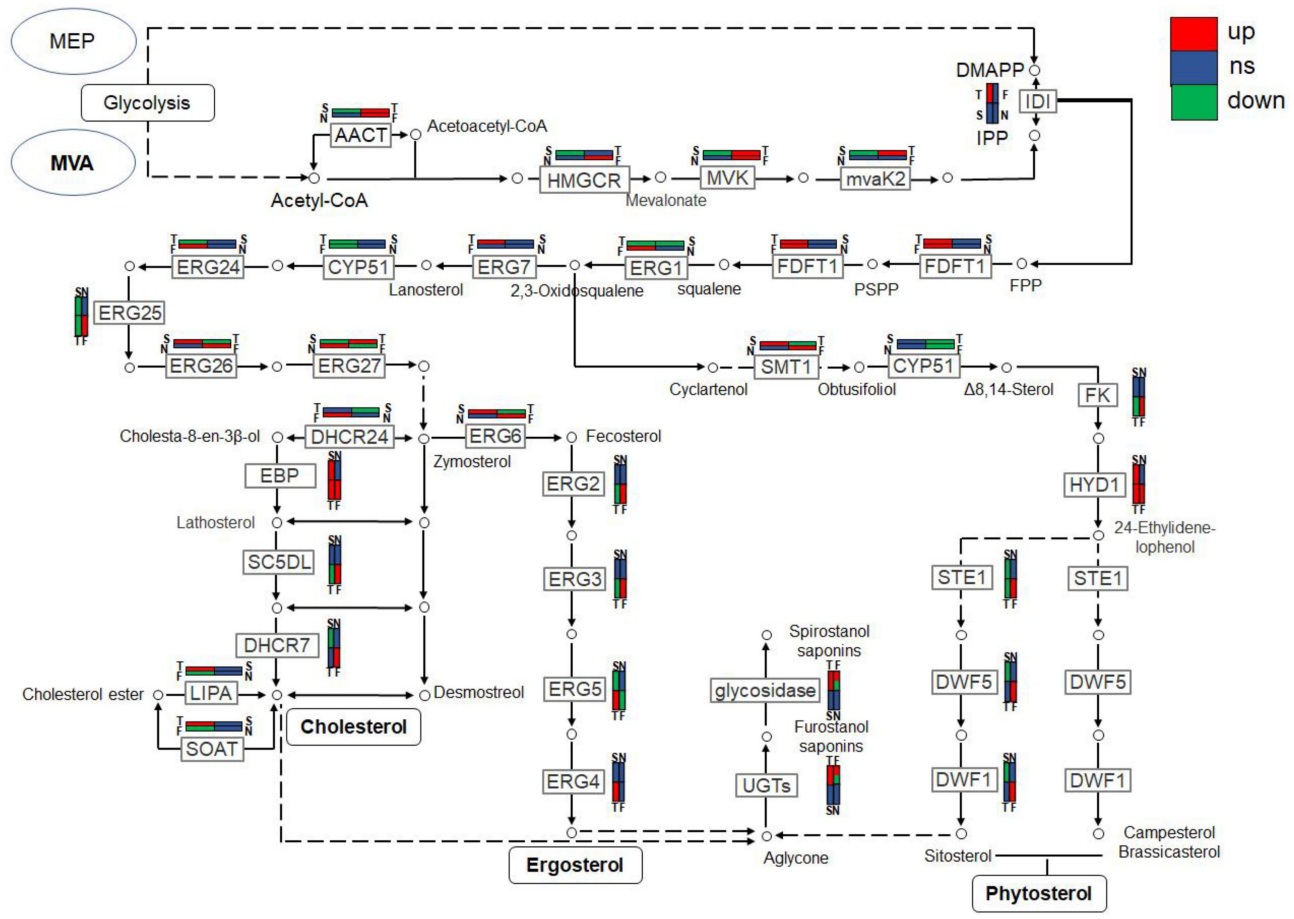
The expression trends of genes related to steroidal saponin synthesis pathway in fermentation on days 3(T), 5(F), 7(S), and 9(N).

Combined with the relevant annotations of the two databases, about 48 DEGs that may be involved in the synthesis and transformation of steroidal saponins were selected (Supplementary Table S2). The synthesis of steroidal saponins can be divided into three stages: sterol synthesis, aglycon synthesis, and saponin synthesis (Chen et al., 2021). In plants, sterol synthesis first relies on the MVA and MEP pathways to synthesize FPP. The screened DEGs showed that strain C39 only relied on the MVA pathway for sterol synthesis, and the DEGs in this process included acetyl-CoA C-acetyltransferase (AACT), Mevalonate kinase (MVK), hydroxymethylglutaryl-CoA reductase (NADPH, HMGCR), phosphomevalonate kinase (mvaK2), and isopentenyl-diphosphate Delta-isomerase (IDI). Then, the obtained FPP synthesizes lanosterol by farnesyl-diphosphate farnesyltransferase (FDFT1), ERG1, and lanosterol synthase (ERG7). Finally, through CYP51 ERG24/FK, methylsterol monooxygenase (ERG25), ERG26, 3-keto steroid reductase (ERG27), cholestenol Delta-isomerase (EPB; HYD1), sterol 24-C-methyltransferase (ERG6; SMT1), C-8 sterol isomerase (ERG2), Delta7-sterol 5-desaturase (ERG3; STE1), sterol 22-desaturase (ERG5), Delta24(24(1))-sterol reductase (ERG4) and Delta24-sterol reductase (DWF1) to synthesize ergosterol, cholesterol or plant sterols. The above-related genes were differentially expressed during the fermentation process. The expression levels of CYP51, ERG2 and ERG25 were the largest in the control group, and the expression levels of other selected DEGs showed a trend of first increase and then decrease or continuous increase on the whole. The expression levels of all DEGs are shown in Figures 11, 12.

The synthesis of saponins also requires the participation of various cytochrome P450s, glycosyltransferases, glycosidases and oxidoreductases. After several steps of cytochrome P450s, sterols are catalyzed into sapogenins, then under the synthesis and modification of glycosyltransferases, glycosidases and some oxidoreductases, saponins with different configurations are produced. By analyzing related genes whose expression levels were significantly increased at D3 or D5, glucosyltransferase, glucosidase, galactosidase and oxidoreductase that may be involved in the synthesis and conversion of steroidal saponins were screened out.

### Validation of DEG profiles by qRT-PCR

**Figure 13:**
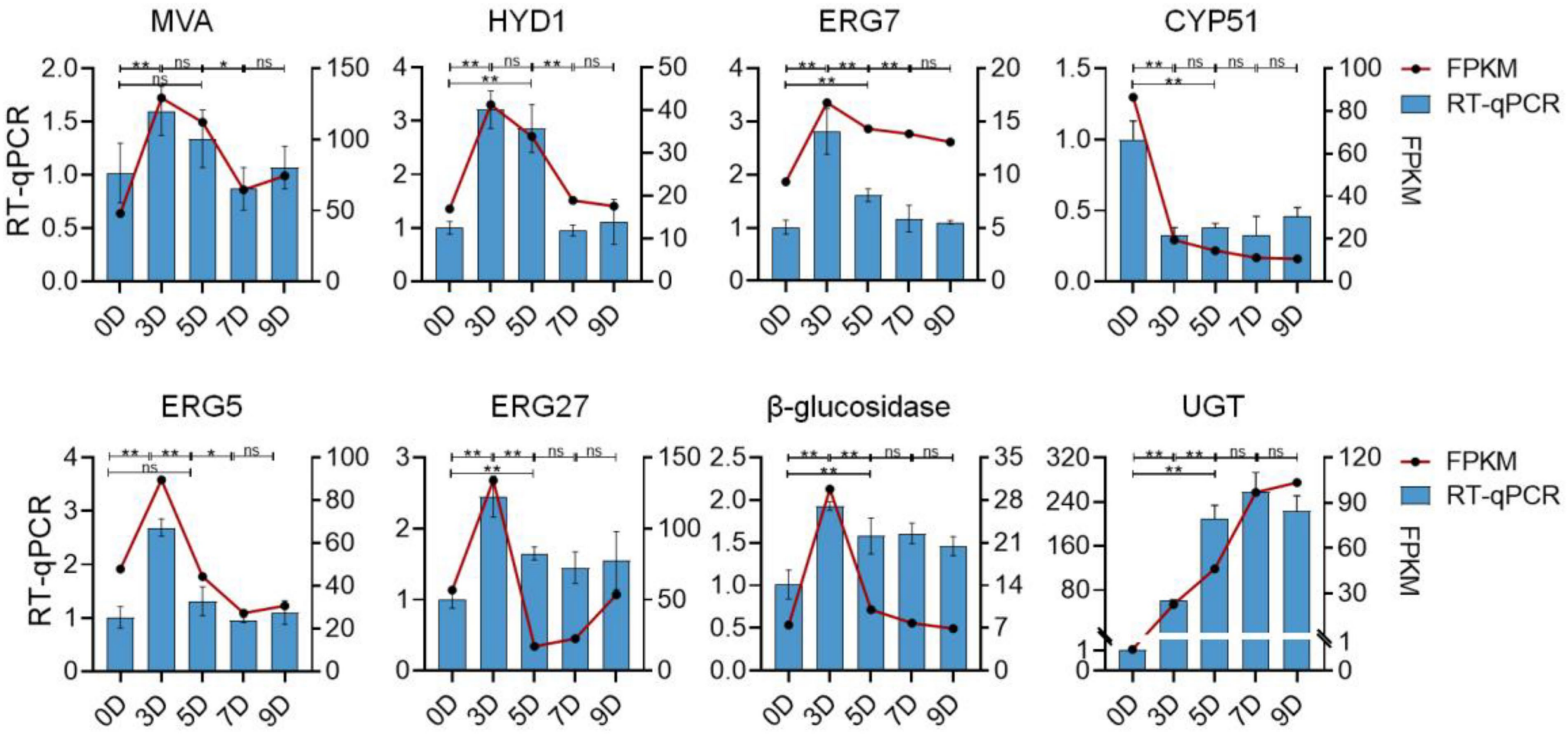
The expression profiles of qRT-PCR and RNA-seq of eight selected genes. The left vertical axis represents the relative expression of the gene based on qRT-PCR. The right vertical axis represents the expression level of the gene based on qRT-PCR. Values are means ± SD. The asterisk above the bar chart denoted statistical significance (*p < 0.05, **p < 0.01, and ns *p* > 0.05).

To validate the reliability of the RNA-Seq data, eight DEGs that may be involved in the promotion and transformation of steroidal saponins were selected for the quantitative real-time PCR (qRT-PCR) analysis. As shown in Figure 13, all the selected genes were differentially expressed under different fermentation days, showing close similarity gene expression trends to RNA-Seq results.

## Discussion

The use of microbial fermentation to synergize, attenuate, or transform medicinal plants for better clinical treatment has a long history. Relying on the microorganism characteristics of high efficiency, strong specificity, economy, and environmental protection, the production and transformation of natural products by microorganisms has become an important way to expand natural medicinal resources, increase the yield of medicinally active ingredients, and find new medicinal substances (Venisetty and Ciddi, 2003; Pham et al., 2019). For example, a variety of pentacyclic triterpenoids were biotransformed into new derivatives by *Nocardia* sp. NRRL 5646 through esterification, methyl transfer, backbone rearrangement, and hydroxylation reactions (Cheng et al., 2004; Zhang et al., 2005; Qian et al., 2009). The main saponins of ginsenosides Rb1, Rb2, Re, and Rg1 in ginseng that is not easy to be directly utilized in the human body were converted by microorganisms into rare saponins with small molecules such as ginsenoside CK, which possess higher bioavailability and better pharmacological activities (Geraldi, 2020). The yield and purity of resveratrol – a precious anti-cancer and antioxidant phenolic substance – are low in natural plants. Using co-immobilized edible *Aspergillus niger* and Yeast, the conversion rate of polydatin can reach 97%, which is significantly higher than that of the control group (Jin et al., 2013). Currently, the main use of steroidal saponins is as the raw material of steroid hormone drugs, but the production process will cause serious environmental pollution (Cheng et al., 2009). Microbial transformation can replace acid hydrolysis to produce diosgenin with high efficiency and environmental protection (Chen et al., 2018). At the same time, microbial biotechnologies play a significant role in the pharmaceutical steroid industry (Donova, 2018). The pharmacological effects of steroidal saponins are closely related to structural characteristics such as sugar chains, glycosyl types, and substituents (Wang et al., 2010; Prawat et al., 2016). Spirostanol saponins have stronger cytotoxicity than furostane-type saponins (Zhao et al., 2009). For spirostanol saponins, diosgenin-type saponins have stronger anti-cancer effects on various cancers, while pennogenin-type saponins show more excellent hemostatic effects (Huang et al., 2007; Fu et al., 2008; Yan et al., 2009). Nuatigenin-type saponins are rare in *Paris polyphylla*, and some show cytotoxicity to human cancer cell lines (HepG2 and HEK293; Qin et al., 2016). Pharmacological studies showed that the fermentation sample had significantly stronger inhibitory effects on the proliferation of Hepal-6 liver cancer cells, Hela cervical cancer cells, and A549 lung cancer cells than its raw medicinal material sample. This proves that strain C39 significantly increased the saponin content and enhanced the anti-cancer effect. There are few studies on the microbial synthesis and promotion of steroidal saponins in *Paris polyphylla*, especially related to the transformation mechanism.

The saponin contents of the four configurations showed different trends during the fermentation process. The content of furostane-type saponins linked to glucose at the C26 position shows an obvious downward trend, while spirostanol saponins increase first. Studies have shown that furostane-type saponins can be converted to spirostanol saponins by breaking the C26 bond and cyclization chain (Nakayasu et al., 2015) suggesting that the biotransformation of furostanol-type saponins to spirostanol saponins occurs under the action of strain C39. *Chaetomium olivaceum* and *Gibberella fujikuroi* are two reported microorganisms with the character of biotransforming pseudoprotodioscin (Dong et al., 2016; Hu et al., 2018). This is the first report of increasing the content of diosgenin-type saponins and pennogenin-type in *Paris polyphylla* by microbial transformation, which promoted the biosynthesis of spirosteroidal saponins with stronger pharmacological activity. Diosgenin can be converted into nuatigenone by microbial biotransformation (Wang et al., 2007). In this study, the content of nuatigenin-type saponins linked to glucose at C26 showed a significant downward trend after 3 days of fermentation and then gradually increased, so it is speculated that this type of saponins may be the intermediates in the biotransformation process. After the cyclization of furostanol saponins, the chemical bond at the C26 position is broken and converted into spirosterol saponins or other types of saponins through isomerization. Peak 2 is a diosgenin-type saponin, which shows an unexpected and obvious decreasing trend. Structurally, it is linked to a glucose saponin at the C23 position, so it is speculated that hydrolysis of glucose at the C23 position may have occurred. The above transformations all depended on the glucose hydrolysis of strain C39. The transformation of microorganisms into natural medicines can also be achieved through other redox reactions. Among the spirosteroidal saponins that have been found, the saponins containing carbonyl or hydroxyl groups show a trend of first decreasing and then increasing or continuing to increase. It is speculated that the diosgenin-type saponins are hydroxylated or carbonylated. This effect was also verified in the study of the mechanism of biotransformation of *Dioscorea nipponica* by strain C39 (Huang et al., 2022). The ability of strain C39 to promote the synthesis and transformation of steroidal saponins in *Paris polyphylla* improved the accumulation of effective substances, supplemented the steroidal saponin synthesis pathway, and improved the utilization of *Paris polyphylla.*

**Figure 14:**
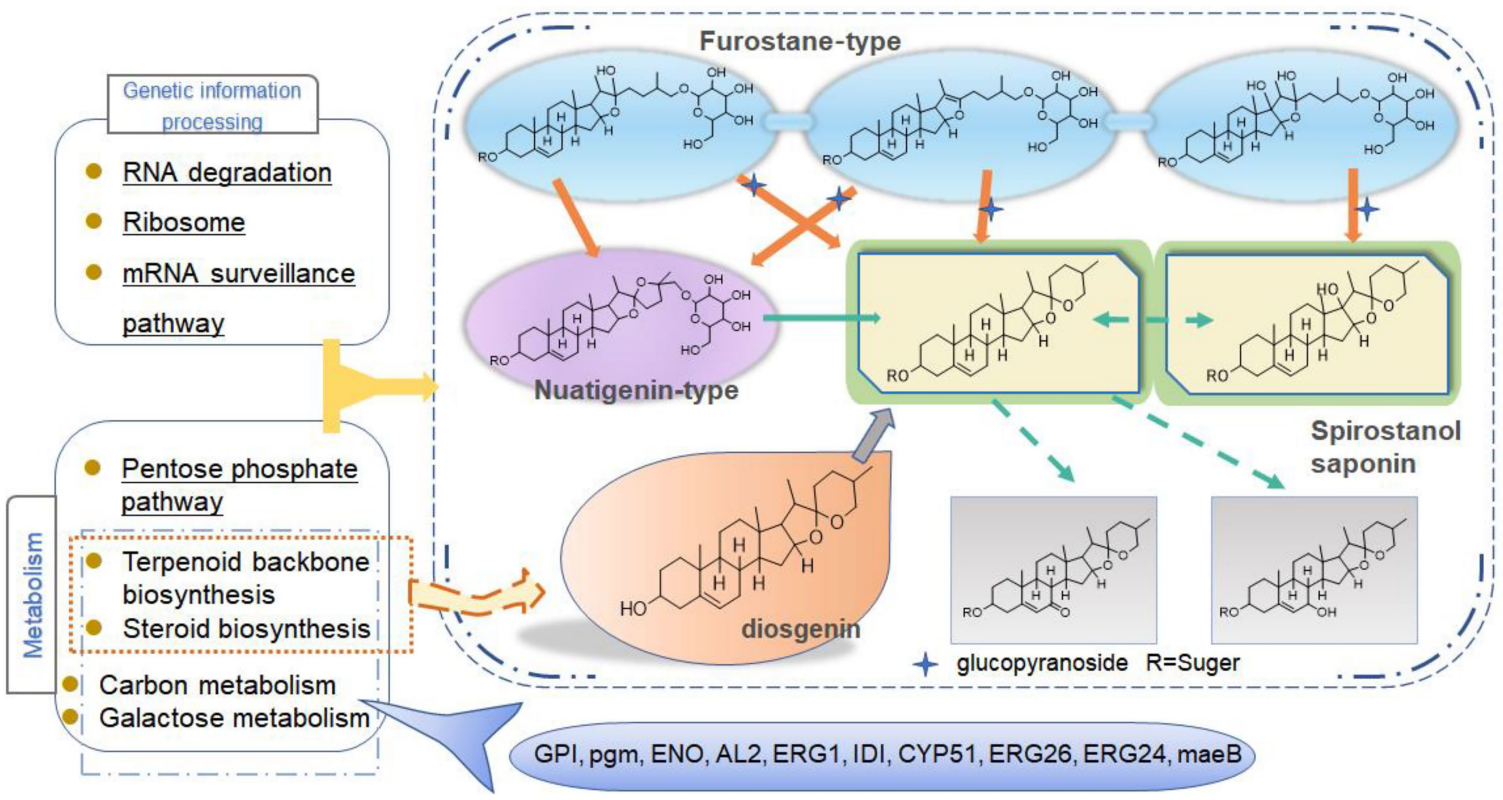
Inferred transformation pathway of strain C39.

To further explain the mechanism of the transformation of steroidal saponins by strain C39, the mycelia of different fermentation times were used for transcriptome analysis. Through GO enrichment analysis, GO terms related to oxidoreductase activity showed a strong correlation. According to the KEGG database, the “Pentose phosphate pathway,” “RNA degradation,” “Ribosome,” and “mRNA surveillance pathway” showed significant enrichment. Hub genes GPI, pgm, ENO, AL2, ERG1, IDI, CYP51, ERG26, ERG24, and maeB, which were involved in “Steroid biosynthesis,” “Glycolysis / Gluconeogenesis,” “Carbon metabolism,” “Terpenoid backbone biosynthesis,” and “Carotenoid biosynthesis,” were considered to be at the key positions in the process of strain C39 increasing the content of steroidal saponins. The sterol required in the synthesis of steroidal saponins is cholesterol or sitosterol, but in fungi, ergosterol occupies a major position, and only certain microorganisms have been found to produce cholesterol or phytosterol (Grandmougin-Ferjani et al., 1999; Wang et al., 2012; Torres et al., 2017). Moreover, most of the genes required for the sterol synthesis pathway of microorganisms are the isoenzymes with those in phytosterols. Studies have shown that the synthesis of cholesterol and diosgenin has been achieved using the sterol synthesis pathway in yeast (Souza et al., 2011; Cheng et al., 2020). The sterol synthesis pathway of strain C39 may be the key pathway for sapogenin synthesis. By screening the expression of related genes in this pathway. None of the related genes on the MEP pathway showed differential expression. However, the expression levels of most genes in the MVA pathway and other processes increased significantly in 3 or 5 days of fermentation, which further verifies this conjecture. The transformation of natural medicines by microorganisms is catalyzed by characteristic enzymes that constantly exhibit substrate promiscuity (Copley, 2015). Designing an *in vitro* synthesis pathway by combining the inherent promiscuous enzyme of protein engineering and metabolic engineering is one of the important solutions to improve the *in vitro* production yield of natural active products. Glycosyltransferase is the key enzyme in converting sapogenin to saponin and the enrichment of saponin species. A glycosyltransferase cloned from *Bacillus subtilis* introduces a glucose at the C-6 position of ginsenoside Rh1 to obtain a potential active saponin (Luo et al., 2015). Sterol glycosyltransferase uses cholesterol, sitosterol, and ergosterol as sugar receptors to synthesize the corresponding sterol glycosides. The sterol glycosyltransferase UGT51 isolated from *Saccharomyces cerevisiae* was used to construct a yeast engineering strain to achieve the maximum titer production of ginsenoside Rh2 synthesized by protopanaxadiol *in vitro* (Zhuang et al., 2017). The conversion of furostanol saponins to spirostanol saponins and the deglycosylation of spirostanol saponins are all completed under glycosidases. The glycosidase PGase-1 isolated and purified from *Aspergillus oryzae* can hydrolyze the terminal 26-O-β-D glucopyranoside and 3-O-(1 → 4)-α-L-rhamnopyranoside (Liu et al., 2013). The β-glucosidase AfG obtained from *Aspergillus fumigatus* has strong thermal stability and can realize the hydrolysis of 3-O-glucopyranoside of various diosgenin-type saponins (Lei et al., 2012). Through transcriptome analysis, seven glycosyltransferases, 11 glycosidases and one oxidoreductase with unknown function were screened that may promote the synthesis of steroidal saponins. These enzymes have potential value for synthesizing or converting of steroidal saponins *in vitro*. The functions of these genes need to be further verified. The inferred transformation pathway of strain C39 is shown in Figure 14.

## Conclusion

In conclusion, this study explored the cytotoxic activity of PRE fermentation products and conducted a preliminary study on how strain C39 increases the steroidal saponins content of Paridis Rhizoma. While strain C39 increased the saponin content, the inhibition of cancer cell proliferation was also enhanced, especially in cervical cancer. Under the action of glycosidase, glycosyltransferase, and oxidoreductase, the configuration and substituent of steroidal saponins were changed. Strain C39 could synthesize diosgenin, and the sterol synthesis pathways were closely related to this process. Further, the synthesized diosgenin may be synthesized into steroidal saponins. The DEGs were significantly enriched in the “Pentose phosphate pathway,” “RNA degradation,” “Ribosome,” and “mRNA surveil-lance pathway,” which means that these four pathways were significantly affected and played a prominent role. Through PPI, hub genes GPI, pgm, ENO, AL2, ERG1, IDI, CYP51, ERG26, ERG24, and maeB were implicated in directly affecting the steroidal saponins content of strain C39. Understanding the synthetic pathway and key genes is the basis for the *in vitro* synthesis of natural products, and exploring the transformation mechanism promoted the *in vitro* synthesis of steroidal saponins in Paridis Rhizoma. In subsequent studies, the functions and properties of key genes in strain C39 involved in regulating steroidal saponin synthesis and transformation will be deeply explored and applied in practice.

## Data availability statement

The datasets presented in this study can be found in online repositories. The names of the repository/repositories and accession number(s) can be found at: https://www.ncbi.nlm.nih.gov/, PRJNA850155.

## Author contributions

YC, XD, and DY conceptualized and designed the experiment. YC performed a study of the transformation mechanism of strain C39 to increase saponin content, analyzed the data, and drafted the manuscript. JH and NH assisted in the analysis of the chemical constituent structures. MZ conducted cancer cell cytotoxicity experiments. XD and DY supervised and edited the manuscript. All authors contributed to the article and approved the submitted version.

## Funding

This research was funded by National Natural Science Foundation of China, grant number 81872967.

## Conflict of interest

The authors declare that the research was conducted in the absence of any commercial or financial relationships that could be construed as a potential conflict of interest.

## Publisher’s note

All claims expressed in this article are solely those of the authors and do not necessarily represent those of their affiliated organizations, or those of the publisher, the editors and the reviewers. Any product that may be evaluated in this article, or claim that may be made by its manufacturer, is not guaranteed or endorsed by the publisher.

## Footnotes

^1^ http://www.geneontology.org/

^2^ https://www.kegg.jp/

^3^ https://en.wikipedia.org/wiki/Hypergeometric_distribution
