# Supplementary material for: Studies on biotransformation mechanism of Fusarium sp. C39 to enhance saponin content of Paridis Rhizoma
Source: Front Microbiol. 2022 Dec 16;13:992318. doi: 10.3389/fmicb.2022.992318 (PMC9800501; doi:10.3389/fmicb.2022.992318)
Supplement: Supplementary file 1 [file Data_Sheet_1.docx]

Supplementary Material

**Table S1.** Primers designed for qRT-PCR.

| **Gene ID** | **Name** | **Forward primer sequence (5'-3')** | **Reverse primer sequence (5'-3')** |
| --- | --- | --- | --- |
| GME763_g | MVA | GCTGGTGGTGGTGGATGTT | TGGCGTAGTTTTCGGCTTC |
| GME6825_g | HYD1 or EPB | GCCTCCCTTCCATGTCTGATGTTG | CGTGCTGCGATTCTTTGCTTGAC |
| GME812_g | ERG7 | CCGCAGCAAAGAAGTCAAAA | AGCAGATACCCCAACTACCATACC |
| GME2055_g | CYP51 | CGGGCAGGTCATCAAGGAAACTC | GAGGCGAGCAAGGTGTGAGATG |
| GME10245_g | ERG5 | TGTCCTTGACCGTGTCCGAGAG | GGCACGAGTGTATGTGAGACCTTC |
| GME7915_g | ERG27 | CGAGACCACCACAAGCACA | GCCAGCCAACAAGCACA |
| GME5623_g | beta-glucosidase | TTCCCAAGACAACAGGCAGA | CCTCCGTCCAGTCCCATAGA |
| GME5092_g | UGT | CCCAAACCGCAACGAAA | TTTTGCCCGCTACTACCACTC |
| GME8911_g | TUBA | AACTCCACCGCCATCCA | GAACTCCATCTCGTCCATACCC |

**Table S2.** Screened genes directly related to steroidal saponin synthesis and conversion.

| **Gene ID** | **Definition** | **Name** | **KEGG enzyme commission（EC）** | **length (bp)** |
| --- | --- | --- | --- | --- |
| GME11738_g | acetyl-CoA C-acetyltransferase | AATC | [EC:2.3.1.9] | 1605 |
| GME11980_g |  |  |  | 858 |
| GME14349_g | alpha-glucosidase | malZ | [EC:3.2.1.20] | 2159 |
| GME9855_g |  |  |  | 2926 |
| GME5623_g | beta-glucosidase | bglX | [EC:3.2.1.21] | 4528 |
| GME2795_g |  |  |  | 2627 |
| GME11483_g |  |  |  | 6992 |
| GME8572_g |  |  |  | 1533 |
| GME6825_g | cholestenol Delta-isomerase | EBP, HYD1 | [EC:5.3.3.5] | 6906 |
| GME14209_g |  |  |  | 5783 |
| GME7294_g |  |  |  | 3661 |
| GME1564_g |  |  |  | 2118 |
| GME8165_g | C-8 sterol isomerase | ERG2 | [EC:5.-.-.-] | 761 |
| GME13425_g | delta14-sterol reductase | ERG24, FK | [EC:1.3.1.70] | 1540 |
| GME12892_g | delta24(24(1))-sterol reductase | ERG4 | [EC:1.3.1.71] | 1956 |
| GME754_g | delta24-sterol reductase | DHCR24, DWF1 | [EC:1.3.1.72 1.3.1.-] | 1506 |
| GME13205_g | delta7-sterol 5-desaturase | ERG3, STE1, SC5DL | [EC:1.14.19.20] | 1090 |
| GME13162_g | farnesyl-diphosphate farnesyltransferase | FDFT1 | [EC:2.5.1.21] | 7525 |
| GME12601_g | geranylgeranyl diphosphate synthase, type III | GGPS1 | [EC:2.5.1.1 2.5.1.10 2.5.1.29] | 1128 |
| GME2073_g | glucan endo-1,3-alpha-glucosidase |  | [EC:3.2.1.59] | 6298 |
| GME4287_g | glucan 1,3-alpha-glucosidase |  | [EC:3.2.1.58] | 2115 |
| GME10777_g |  |  |  | 1476 |
| GME14352_g | hydrolase activity |  |  | 1824 |
| GME5692_g | hydroxymethylglutaryl-CoA reductase | HMGCR, NADPH | [EC:1.1.1.34] | 4113 |
| GME5989_g | hydroxymethylglutaryl-CoA synthase | HMGCS | [E2.3.3.10] | 1834 |
| GME6440_g | isopentenyl-diphosphate Delta-isomerase | IDI | [EC:5.3.3.2] | 4212 |
| GME812_g | lanosterol synthase | ERG7 | [EC:5.4.99.7] | 2432 |
| GME13737_g | lysosomal acid lipase/cholesteryl ester hydrolase | LIPA | [EC:2.4.1.267] | 2130 |
| GME6325_g | methylsterol monooxygenase | ERG25, MESO1 | [EC:1.14.18.9] | 1053 |
| GME763_g | mevalonate kinase | MVK, mvaK1 | [EC:2.7.1.36] | 3766 |
| GME6418_g | phosphomevalonate kinase | mvaK2 | [EC:2.7.4.2] | 6533 |
| GME6592_g | squalene monooxygenase | ERG1, SQLE | [EC:1.14.14.17] | 1503 |
| GME12919_g | sterol O-acyltransferase | SOAT | [EC:2.3.1.26] | 2227 |
| GME10420_g |  |  |  | 1928 |
| GME 1464_g | sterol 3beta-glucosyltransferase |  | [EC:2.4.1.173] | 7522 |
| GME5092_g |  |  |  | 2841 |
| GME7373_g |  |  |  | 2872 |
| GME11674_g |  |  |  | 4040 |
| GME4321_g |  |  |  | 5367 |
| GME2793_g | sterol-4alpha-carboxylate 3-dehydrogenase | ERG26, NSDHL | [EC:1.1.1.170] | 1003 |
| GME13615_g |  |  |  | 960 |
| GME14223_g |  |  |  | 4014 |
| GME2055_g | sterol 14alpha-demethylase | CYP51 | [EC:1.14.14.154] | 1641 |
| GME3477_g |  |  |  | 1727 |
| GME10245_g | sterol 22-desaturase | ERG5, CYP61A | [EC:1.14.19.41] | 3732 |
| GME9119_g | sterol 24-C-methyltransferase | ERG6, SMT1 | [EC:2.1.1.41] | 897 |
| GME7915_g | 3-keto steroid reductase | ERG27 | [EC:1.1.1.270] | 983 |
| GME5127_g | 7-dehydrocholesterol reductase | DWF5, DHCR7 | [EC:1.3.1.21] | 954 |

| **(A)** | **(B)** | **(C)** |
| --- | --- | --- |

**Figure S1.** GO terms analysis of DEGs during the fermentation of **(A)** D3-vs-D5, **(B)** D5-vs-D7 and **(C)** D7-vs-D9.


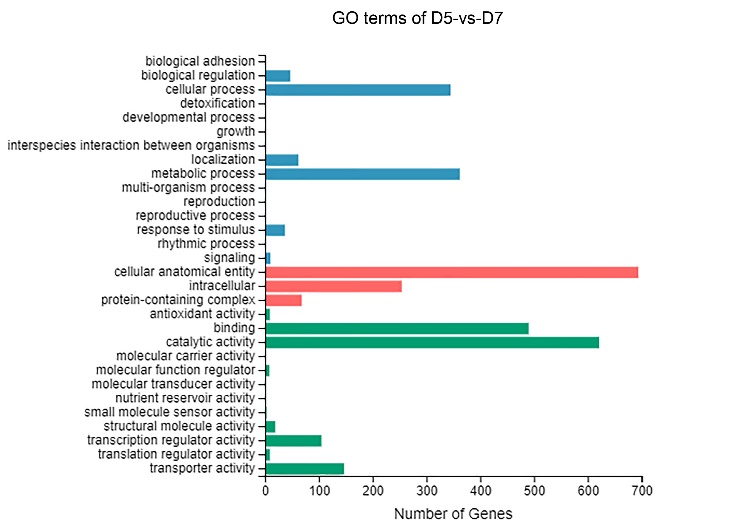

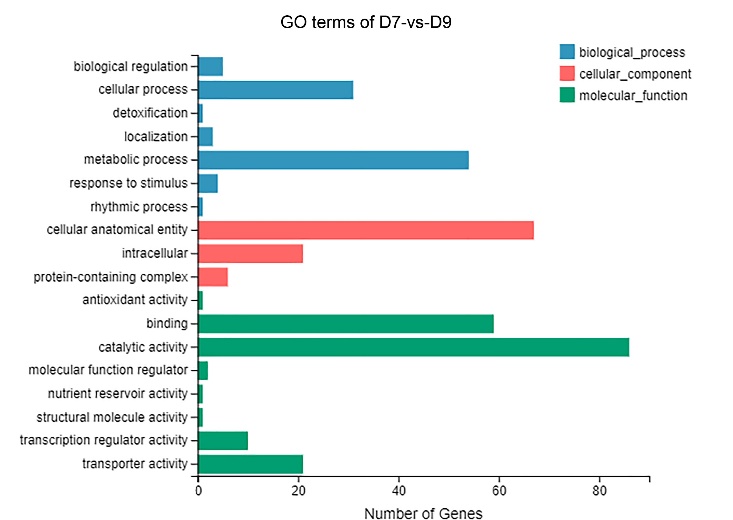

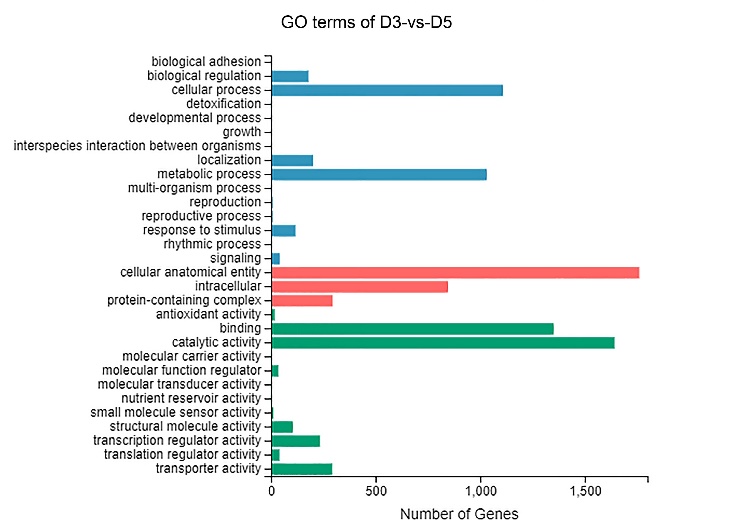

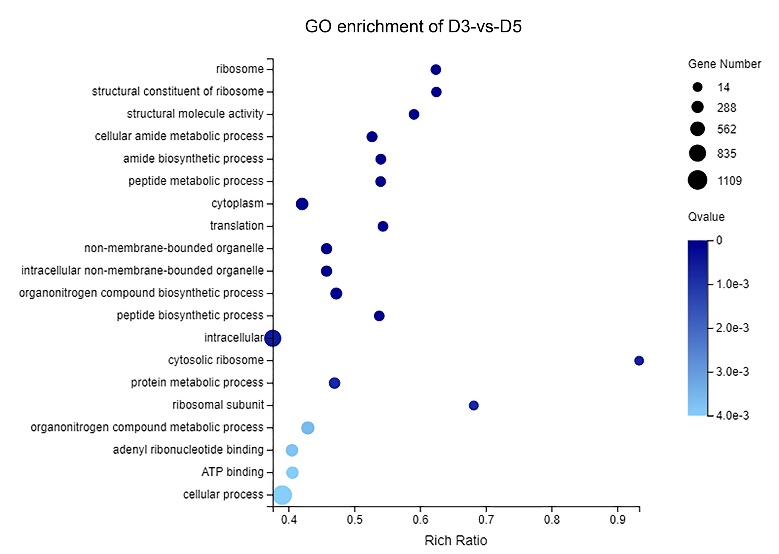

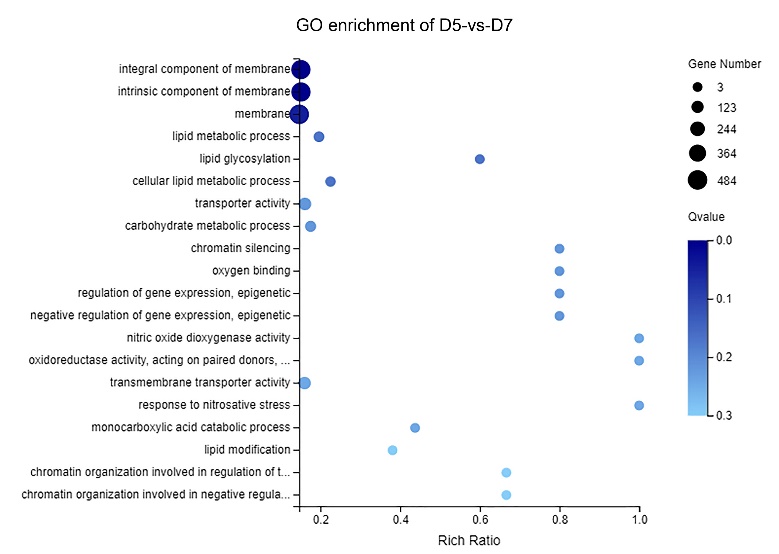

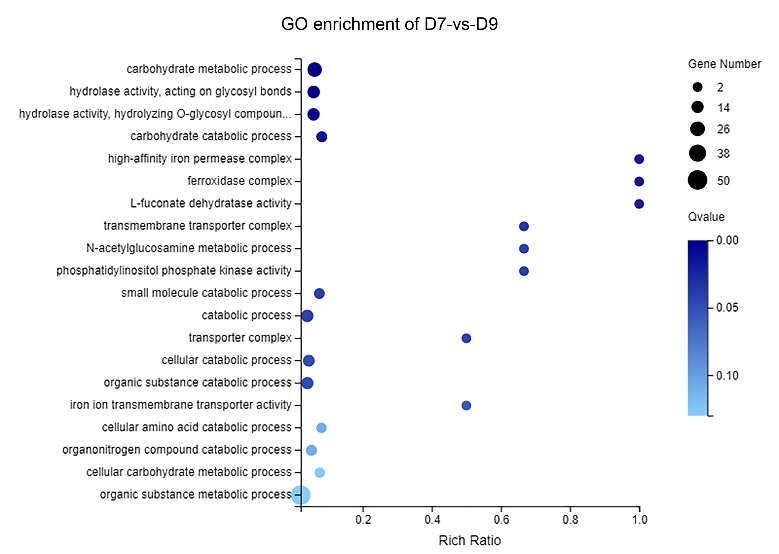


| **(A)** | **(B)** | **(C)** |
| --- | --- | --- |

**Figure S2**. GO enrichment analysis of DEGs during the fermentation of **(A)** D3-vs-D5, **(B)** D5-vs-D7 and **(C)** D7-vs-D9, showing the top 20 GO terms with the smallest Q values.

| **(A)** | **(B)** | **(C)** |
| --- | --- | --- |

**Figure S3**. KEGG pathway terms of DEGs during the fermentation of **(A)** D3-vs-D5, **(B)** D5-vs-D7 and **(C)** D7-vs-D9.


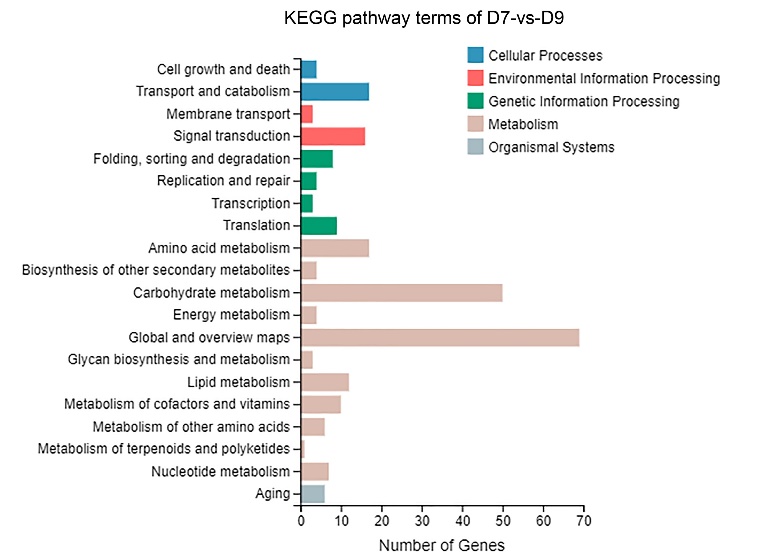

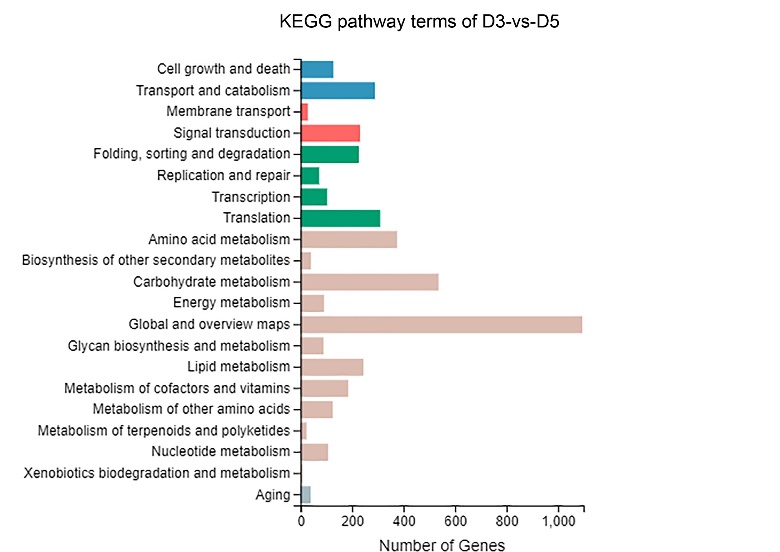

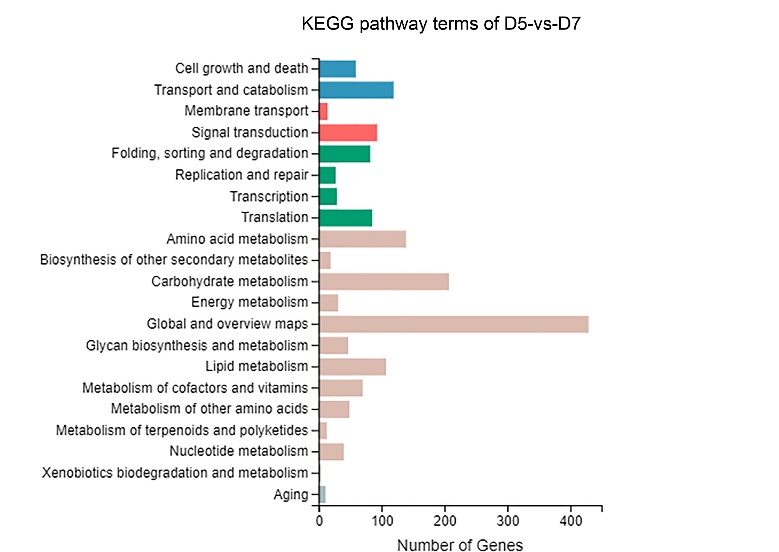

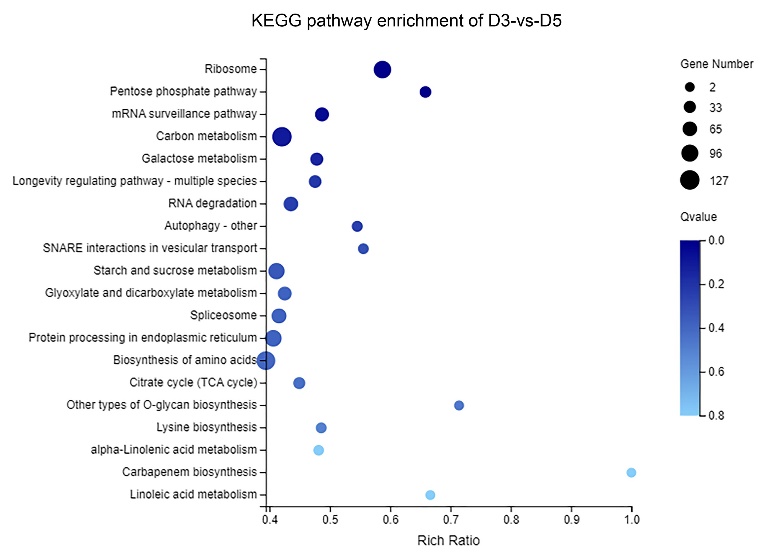

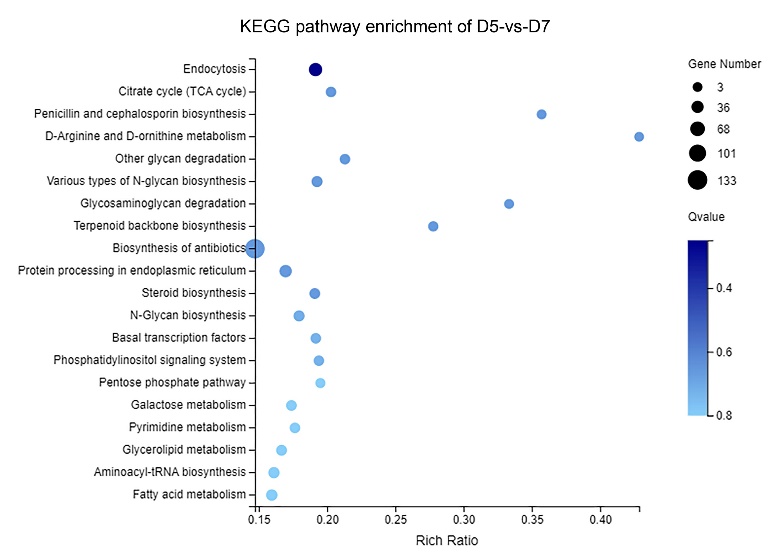

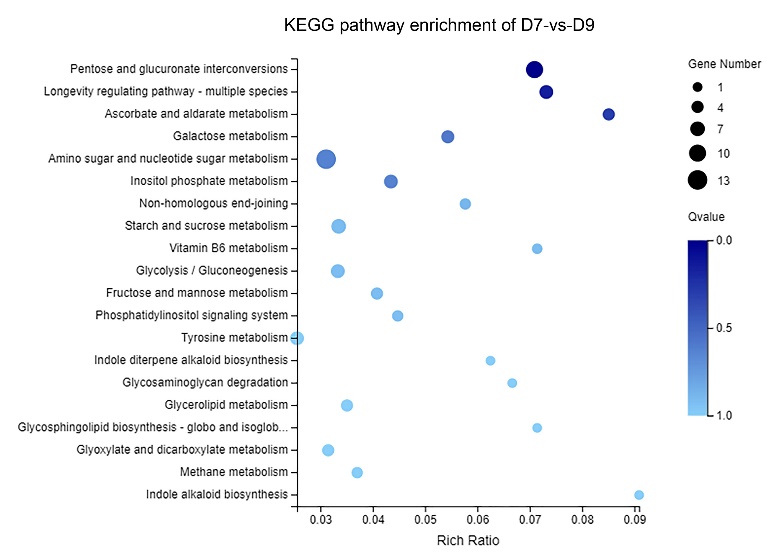


| **(A)** | **(B)** | **(C)** |
| --- | --- | --- |

**Figure S4.** KEGG pathway enrichment analysis of DEGs during the fermentation of **(A)** D3-vs-D5, **(B)** D5-vs-D7 and **(C)** D7-vs-D9 showing the top 20 GO terms with the smallest Q value.
